# Protective Role of Dietary Polyphenols in the Management and Treatment of Type 2 Diabetes Mellitus

**DOI:** 10.3390/nu17020275

**Published:** 2025-01-13

**Authors:** Monika Martiniakova, Anna Sarocka, Noemi Penzes, Roman Biro, Veronika Kovacova, Vladimira Mondockova, Aneta Sevcikova, Sona Ciernikova, Radoslav Omelka

**Affiliations:** 1 Department of Zoology and Anthropology, Faculty of Natural Sciences and Informatics, Constantine the Philosopher University in Nitra, 94901 Nitra, Slovakia; roman.biro@ukf.sk (R.B.); vkovacova@ukf.sk (V.K.); 2 Department of Botany and Genetics, Faculty of Natural Sciences and Informatics, Constantine the Philosopher University in Nitra, 94901 Nitra, Slovakia; asarocka@ukf.sk (A.S.); noemi.penzes@ukf.sk (N.P.); vmondockova@ukf.sk (V.M.); 3 Department of Genetics, Cancer Research Institute, Biomedical Research Center of the Slovak Academy of Sciences, 84505 Bratislava, Slovakia; aneta.sevcikova@savba.sk (A.S.); sona.ciernikova@savba.sk (S.C.)

**Keywords:** type 2 diabetes mellitus, flavonoids, phenolic acids, tannins, stilbenes, lignans, antidiabetic drugs, polyphenol-antidiabetic drug interactions

## Abstract

Type 2 diabetes mellitus (T2DM), a serious metabolic disorder, is a worldwide health problem due to the alarming rise in prevalence and elevated morbidity and mortality. Chronic hyperglycemia, insulin resistance, and ineffective insulin effect and secretion are hallmarks of T2DM, leading to many serious secondary complications. These include, in particular, cardiovascular disorders, diabetic neuropathy, nephropathy and retinopathy, diabetic foot, osteoporosis, liver damage, susceptibility to infections and some cancers. Polyphenols such as flavonoids, phenolic acids, stilbenes, tannins, and lignans constitute an extensive and heterogeneous group of phytochemicals in fresh fruits, vegetables and their products. Various in vitro studies, animal model studies and available clinical trials revealed that flavonoids (e.g., quercetin, kaempferol, rutin, epicatechin, genistein, daidzein, anthocyanins), phenolic acids (e.g., chlorogenic, caffeic, ellagic, gallic acids, curcumin), stilbenes (e.g., resveratrol), tannins (e.g., procyanidin B2, seaweed phlorotannins), lignans (e.g., pinoresinol) have the ability to lower hyperglycemia, enhance insulin sensitivity and improve insulin secretion, scavenge reactive oxygen species, reduce chronic inflammation, modulate gut microbiota, and alleviate secondary complications of T2DM. The interaction between polyphenols and conventional antidiabetic drugs offers a promising strategy in the management and treatment of T2DM, especially in advanced disease stages. Synergistic effects of polyphenols with antidiabetic drugs have been documented, but also antagonistic interactions that may impair drug efficacy. Therefore, additional research is required to clarify mutual interactions in order to use the knowledge in clinical applications. Nevertheless, dietary polyphenols can be successfully applied as part of supportive treatment for T2DM, as they reduce both obvious clinical symptoms and secondary complications.

## 1. Introduction

Diabetes mellitus (DM), a serious non-communicable disease, represents a global health problem. It is regarded as a chronic metabolic disturbance that is unfortunately on the rise and is distinguished by hyperglycemia due to inadequate insulin production, altered insulin action, or a combination thereof [1,2]. Globally, 537 million people had DM in 2021. Type 2 DM (T2DM), also referred to as non-insulin-dependent diabetes, is the most prevalent type of the disease, accounting for approximately 90% of all cases. Glycated hemoglobin (HbA1c) criteria and fasting or 2-h plasma glucose criteria are typically used to diagnose T2DM. It is anticipated that 592 million people worldwide will have T2DM by 2035, with up to 374 million subjects at increased risk of developing this disease [3,4]. Since about 80% of people with T2DM reside in states with low and middle incomes, the prevalence of this disease is linked to economic disparities [5]. It is widely recognized that obesity, poor diet, physical inactivity, damaged mental health, and genetic predispositions are responsible for T2DM progression [4,6]. The main biological hallmark of T2DM is insulin resistance, which is related to elevated levels of reactive oxygen species (ROS), resulting in chronic oxidative stress and inflammatory process. Superoxide anion radical, hydroxyl radical, singlet oxygen, and hydrogen peroxide are among the most common ROS [7]. Furthermore, oxidized low-density lipoprotein (LDL) cholesterol, which is linked to the development of atherosclerotic plaque, can also be formed by an excess of ROS. Common comorbidities in T2DM include atherosclerosis and associated cardiovascular disorders [4,5]. Diabetic neuropathy, nephropathy and retinopathy, diabetic foot, osteoporosis (also termed as diabetic bone disease), liver damage, susceptibility to infections and some cancers (e.g., pancreas, liver, colon, endometrium, breast, bladder) are additional severe long-term complications of T2DM [8,9,10]. Increased oxidative stress, which results from an imbalance between ROS production and the antioxidant defense system (ADS), is also the cause of all of the secondary complications mentioned above [11]. The ADS is generally essential for preserving cellular redox homeostasis and scavenging ROS. Among its essential components are glutathione peroxidase (GPx), catalase (CAT), and superoxide dismutase (SOD), as well as sirtuins and peroxisome proliferator-activated receptor-gamma (PPAR-γ) [12].

T2DM manifests itself through two main pathological defects at the molecular level, i.e., disturbed insulin secretion due to pancreatic β-cell dysfunction and defective effect of insulin due to insulin receptor abnormalities [13]. Dysfunction of pancreatic β-cells is affected by endoplasmic reticulum stress and mitochondrial dysfunction. In the diabetic stage, there is a disorder of hepatic glucose production. In the liver and peripheral tissues, the effects of insulin are lessened, as evidenced by increased hepatic gluconeogenesis and glycogenolysis, decreased peripheral tissue uptake of glucose, and elevated blood glucose levels [10,14]. Insulin receptors belong to a family of receptors with tyrosine kinase activity. Upon binding of insulin to its receptor, conformational alterations of the receptor and autophosphorylation occur, along with the initiation of its tyrosine kinase activity. These changes are associated with tyrosine phosphorylation of insulin receptor substrate (IRS) proteins, which trigger intracellular signalling cascades [15,16]. As a result of the activation of the phosphatidylinositol-3-kinase (PI3K)/protein kinase B (PKB or Akt) pathway, insulin or insulin-like growth factor (IGF)-1 are activated. Gene expression and mitogenic effects related to insulin are regulated through the mitogen-activated protein kinase/Ras pathway [16]. Insulin is also able to enhance glucose uptake into the target (e.g., skeletal muscle, liver, adipose) tissues by activating the PI3K/Akt signalling pathway, regulating the transport of glucose transporter type 4 (GLUT4) from intracellular compartments to the surface of the cell membrane [5,15]. GLUT4 then imports glucose into the cell [17].

By modifying intracellular signalling pathways, enhancing the ADS, and neutralizing ROS, nutritional antioxidants found in a range of food sources have demonstrated encouraging potential in lowering oxidative stress and enhancing glycemic control [18,19]. In foods of plant origin, polyphenols constitute an extensive and heterogeneous group of phytochemicals. They are concentrated in fresh fruits, vegetables, seeds, spices, and beverages (e.g., tea, wine, coffee). Additionally, they exhibit powerful antioxidant and anti-inflammatory characteristics, and the consumption of foods rich in polyphenols is linked to a low prevalence of metabolic conditions including obesity, T2DM, and hypertension [10,20,21]. The classification commonly used in the medical literature divides polyphenols into non-flavonoid polyphenols and flavonoids [22]. On the basis of their carbon skeleton, non-flavonoid polyphenols can be classified into different subgroups. Phenolic acids, stilbenes, tannins, and lignans are among the most famous. The flavonoids include primarily flavanones, flavones, dihydroflavonols, flavonols, flavan-3-ols, isoflavones, anthocyanidins, and chalcones [23].

Summarizing the current understanding of dietary polyphenols, emphasizing their protective function in the management and treatment of T2DM, and outlining potential mechanisms of action were the primary goals of this review. In this context, detailed characterizations of flavonoids, phenolic acids, stilbenes, tannins, and lignans were conducted. Biosynthesis, metabolism, and the key conclusions from in vitro and in vivo studies suggesting their antidiabetic potential were discussed in each of the previously mentioned groups. Our primary focus was on available clinical trials. To make this review even more comprehensive, interactions between plant-derived polyphenols and antidiabetic drugs were also included.

## 2. Polyphenols as Antidiabetic Agents

Recent studies suggest that dietary polyphenols have a significant role in the management of T2DM through both insulin-dependent and insulin-independent mechanisms. Insulin-dependent approaches include protecting pancreatic β-cells and promoting their proliferation, reducing pancreatic β-cell apoptosis, alleviating oxidative stress, activating insulin signalling, and stimulating insulin secretion. Mechanisms independent of insulin involve inhibition of glucose absorption, digestive enzymes and advanced glycation end products (AGEs) formation, modification of the inflammatory response, and regulation of gut microbiota. In addition, dietary polyphenols are able to mitigate several serious secondary complications of T2DM [24,25].

More specifically, polyphenols moderate insulin resistance through several mechanisms. They can serve as inhibitors of IRS protein phosphorylation, promoters of GLUT4 translocation, enhancers of Akt phosphorylation, and efficient ROS scavengers [17]. Polyphenols have been shown to improve GLUT4 translocation by the activation of Rab proteins (GTPases and regulators of vesicular transport [26]) and insulin, PI3K, and adenosine monophosphate-activated protein kinase (AMPK) pathways [27,28]. The antioxidant activity of polyphenols may involve direct scavenging of free radicals by hydrogen atom or single electron transfer, or may act through a transition metal chelation mechanism [29], which activates also the action of antioxidant enzymes [1]. Polyphenols are able to protect pancreatic β-cells from oxidative stress both through antioxidant effects and by activating anti-apoptosis signalling [30]. Considering the inflammatory response, polyphenols suppress the production of pro-inflammatory cytokines, including interleukin (IL)-6, IL-8, tumour necrosis factor-alpha (TNF-α) via the inhibited activation of mitogen-activated protein kinases (MAPKs) and nuclear factor kappa B (NF-κB) pathways, and subsequently alleviate inflammatory processes [5]. They stimulate insulin secretion through upregulation of AMPK and IRS pathways and lower oxidative damage to pancreatic β-cells, thus preserving β-cell integrity [31]. Stimulation of insulin release probably occurs through transient inhibition of ATP-sensitive K^+^ channels and stimulation of whole-cell Ca^2+^ [17]. Polyphenols also modulate hepatic glucose production by upregulating carnitine palmitoyltransferase 1-β and acyl-CoA oxidase 1 and downregulating phosphoenolpyruvate carboxylase and glucose-6 phosphatase [32]. They further serve as inhibitors of carbohydrate-digesting enzymes and glucose absorption by interacting with α-amylase, α-glucosidase, and sodium-dependent glucose transporter 1 (SLGT1) [33]. Moreover, they activate glucose uptake receptors in insulin-sensitive tissues [34].

Mounting evidence suggests that gut dysbiosis, characterized by a disrupted intestinal balance and an increased prevalence of unfavourable gut microorganisms, plays a significant role in T2DM [35]. Higher levels of *Escherichia* and *Prevotella* have been detected in T2DM patients, whereas beneficial bacteria such as *Bifidobacterium* and *Roseburia* are more abundant in healthy individuals [36]. At the phylum level, the Firmicutes to Bacteroidetes ratio is elevated in patients compared to non-diabetic controls [37]. It is widely recognized that short-chain fatty acids (SCFAs), the major end products of bacterial fermentation in the gut, may also play a key role in the etiology of T2DM [38,39]. Data indicate that butyrate-producing bacterial taxa are significantly reduced in T2DM subjects [40]. Butyrate, a SCFA produced by gut bacteria in the colon, can prevent the development of insulin resistance [41,42,43,44]. Therefore, gut microbiota modulation is crucial for restoring intestinal balance, improving insulin sensitivity and glucose metabolism, and reducing the risk of DM-associated complications.

Polyphenols and their microbiota-derived metabolites can effectively modulate gut microbiota balance and improve glucose metabolism by promoting the growth of beneficial bacteria, including *Bifidobacteria*, *Akkermansia*, and *Faecalibacterium prausnitzii* [45]. Additionally, polyphenols exert antidiabetic, antihypertensive, and anti-inflammatory activities [46]. Supplementation with prebiotics such as inulin, galactooligosaccharides, fructooligosaccharides, pectic oligosaccharides, starch, polyphenols, and β-glucan, combined with *Dendrobium officinale*, has been shown to promote glycemic control in T2DM [47]. An in vitro study demonstrated that oregano polyphenols might be used as hypoglycemic and hypolipidemic agents [48]. Numerous animal studies on T2DM have confirmed the favourable impacts of polyphenols in remodelling gut microbiota, which may contribute to improvements in this chronic condition. Tea polyphenols, for instance, counteracted gut dysbiosis and reduced both central and peripheral inflammation in a T2DM rat model. Furthermore, tea polyphenols alleviated memory impairments, potentially through gut microbiota-mediated attenuation of neuroinflammation via inhibition of the TLR4/NF-κB pathway [49]. A review of 11,400 participants with T2DM documented that tea consumption (≥4 cups/day) might play a role in the prevention of T2DM and reduce the risk of disease [50].

Oral gavage of rambutan peel polyphenols improved glucolipid metabolism and altered gut microbiota, particularly the levels of *Lactobacillus*, *Tuzzerella*, *Odoribacter*, *Turicibacter*, *Erysipelatoclostridium*, and *Lachnospiraceae* NK4A136 group in a murine model of T2DM [51]. The extract of *Pueraria thomsonii* Radix containing nine natural polyphenols reduced pancreatic tissue damage and decreased the Firmicutes/Bacteroidetes ratio in the T2DM murine model [52]. Polyphenols in vinegar extract restored gut composition and upregulated levels of *Lactobacillus*, *Bifidobacterium*, Bacteroidetes, and *Bacteroides* in a diabetic mouse model. Moreover, the intervention decreased blood glucose and inflammation [53]. Punicalagin, a phenolic compound from pomegranates, improved diabetic renal injury, restored gut composition, and increased cecal SCFA levels in mice [54]. Recently, Li et al. [55] investigated that mulberry polyphenols elevated the abundance of Bacteroidetes and increased butyrate and propionate production in diabetic mice. Moreover, this supplementation enhanced glucose homeostasis via increased glucose utilization, reduced pancreatic damage, and elevated antioxidant capacity. Blueberry juice enriched in polyphenols improved glucose tolerance in a prediabetic mouse model [56]. Zuo et al. [57] assessed the effect of metallothionein-kidney bean polyphenol complex on gut microbiota and glucose levels in diabetic rats. The extraction of polyphenol from kidney beans reversed gut dysbiosis and elevated SCFA levels. According to Sun et al. [24], polyphenols from guava tea, coffee, cocoa, olive oil, propolis, red wine, chocolate, blueberries, and grape seeds have demonstrated antidiabetic impacts in T2DM patients by enhancing glucose metabolism, lowering insulin resistance, HbA1c, and ameliorating vascular function. The impact of flavonoids, phenolic acids, stilbenes, tannins, and lignans in relation to T2DM will be presented in the following chapters.

### 2.1. Flavonoids and T2DM

**Figure 1:**
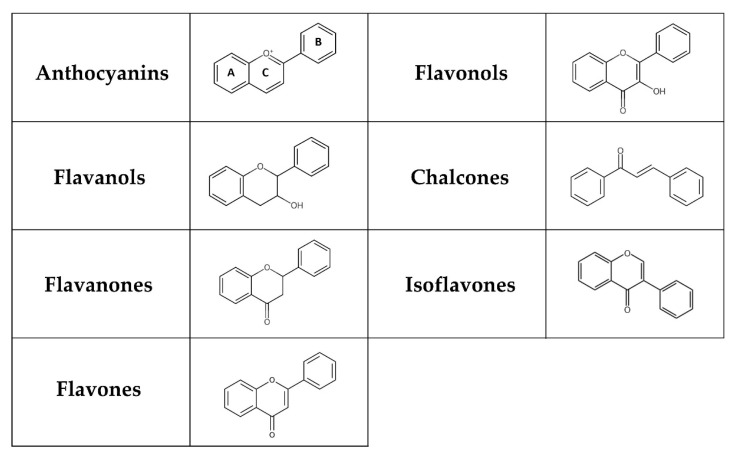
Chemical structure of flavonoids. Flavonoids possess a basic 15-carbon flavone skeleton, with two benzene rings (A and B) linked by a three-carbon pyran ring (C). Flavonols differ from flavones by the presence of a hydroxyl group at the C-3 position, whereas they differ from flavanones by the presence of a hydroxyl group at the C-3 position and a double bond between C-2 and C-3. Flavanols (flavan-3-ols) are 3-hydroxy derivatives of flavanones lacking a double bond between C-2 and C-3. A charged oxygen atom in ring C and the open form of ring C is found in anthocyanins and chalcones, respectively. In isoflavones, the hydrogen at the C-3 position instead of C-2 is replaced by a phenyl group.

Flavonoids represent a group of polyphenols derived from the benzo-γ-pyrone structure [58]. They are produced through the phenylpropanoid pathway in plants as secondary metabolites, playing a significant role in plant defence mechanisms and the colouration of fruits and flowers [59,60,61]. Flavonoids can be classified into flavones, flavanones, isoflavones, flavonols, chalcones, flavanols, and anthocyanins depending on their chemical structure, degree of hydroxylation and polymerization, substitutions and conjugations (Figure 1) [62,63]. Multiple hydroxyl groups in the flavonoid skeleton offer targets for glycosylation, and thus, dietary flavonoids are mostly present in a chemical structure containing O-glycosides or C-glycosides, including mainly glucose, galactose, arabinose, and rhamnose [63]. The flavonoid glycosides exert a low bioavailability that varies with the location and structure of the glycoside. Furthermore, absorption depends on many factors, such as dosage, diet, sex, and the microbial population in the large intestine. While some flavonoids, like anthocyanins, can be absorbed in the stomach, most anthocyanins primarily reach the colon, where they interact with various bacterial communities [64].

Flavonoid glucosides, including quercetin 3-O-glucoside and quercetin 4′-O-glucoside, are able to be absorbed unaltered in the small intestine through sodium-glucose linked transporter 1 (SGLT1) [65]. An additional method to increase flavonoid glucosides’ bioavailability is their hydrolysis to aglycones by α-glucosidase [66]. Another absorption mechanism in the small intestine is represented by lactase phlorizin hydrolase, which is involved in the hydrolysis of flavonoid glycosides [67]. Finally, unabsorbed glycosides enter the large intestine where they are modified by the gut microbiota into corresponding aglycones and absorbed by epithelial cells into the bloodstream [68]. The pyrone ring in the flavonoid is degraded by gut microbiota, resulting in the formation of phenylacetic acid, phenylpropionic acid and inert by-products. Latterly, flavonoids undergo methylation, hydroxylation, O-methylation, sulfation, and glucuronidation, leading to their excretion in urine and faeces [69].

Tea and wine (mainly red wine) are the main dietary sources of flavonoids in Eastern and Western societies. In addition, vegetables (e.g., lettuce, kale, broccoli, onions, celery, parsley, tomatoes), citrus fruits (e.g., lemons, oranges, grapes, grapefruit), apples, bananas, cherries, peaches, berries (e.g., blueberries, strawberries), soybeans, medicinal plants (e.g., Ginkgo biloba, chamomile, mint), cocoa, dark chocolate are considered important sources of dietary flavonoids [70,71,72].

Numerous flavonoids and extracts rich in flavonoids have been studied as potential antidiabetic agents in clinical, animal, and in vitro studies. Considering total flavonoid consumption, a meta-analysis of prospective cohort studies by Liu et al. [73] demonstrated an association between higher total flavonoid intake and reduced risk of T2DM in a dose-dependent manner. In an observational cohort study of 3430 nondiabetic participants, a 33% reduction in new-onset diabetes was observed in the highest versus the lowest tertile of total flavonoid intake and inverse association with T2DM risk was also found for dihydroflavonols and flavanones after 5.51 years of follow-up [74]. Regarding the effect of specific flavonoids on T2DM, a single oral dose (400 mg) of quercetin, one of the most widespread flavonoids, significantly lowered postprandial blood glucose levels in T2DM patients loaded with maltose (2 g/kg), which could be explained by the inhibition of α-glucosidase [75]. Unfortunately, this study included only 12 individuals per group. However, a longer study conducted by Lee et al. [76] that lasted 10 weeks and included 92 healthy male smokers showed improvements in blood glucose levels, lipid profiles, and blood pressure in subjects who received 100 mg of quercetin each day. Many animal experiments have confirmed the hypoglycemic properties of quercetin, where doses of 15–100 mg/kg for 14–70 days resulted in significant reductions in blood glucose levels, particularly by regeneration of pancreatic islets, improving insulin resistance, affecting glucose metabolism, and promoting insulin release [25,77]. According to the findings, 10 weeks of quercetin supplementation lowered body weight and decreased serum insulin level in diabetic mouse model. Data showed that quercetin reduced the amount of *Bacteroides*, *Proteobacteria*, *Escherichia coli*, and *Escherichia-Shigella* and decreased the levels of metabolites, including 3-methoxytyramine, L-aspartic acid, L-glutamic acid, and androstenedione [78]. Quercetin is distinguished also by its anti-inflammatory and antioxidant activity. At the molecular level, quercetin increased the expression of insulin-signalling molecules, such as PI3K, and IRS-1. It also activated AMPK and suppressed NF-κB and Jun *N*-terminal kinase (JNK) pathways [79,80]. In vitro analyses, in silico models and molecular docking studies revealed that quercetin efficiently inhibits α-glucosidase and α-amylase by creating hydrogen bonds with particular amino acid residues [81]. Similarly, kaempferol is considered as an α-glucosidase and α-amylase inhibitor [82]. Furthermore, oral administration of kaempferol improved fasting hyperglycemia, glucose intolerance, and insulin resistance in diet-induced obese mice by inhibiting hepatic gluconeogenesis (reduced pyruvate carboxylase and glucose-6 phosphatase activity) and improving hepatic glucose metabolism (increased Akt and glucose activity) [83]. Bai et al. [84] evaluated the impact of mulberry leaf flavonoids including kaempferol, quercetin, rhamnocitrin, tetramethoxyluteolin, and norartocarpetin on T2DM. They found that kaempferol is capable of binding to protein tyrosine phosphatase 1B (PTP1B) and could be involved in the inhibition of PTP1B.

A randomized clinical trial with a placebo control including 50 participants with T2DM demonstrated that taking 1 g supplement tablet containing 500 mg of rutin daily for 3 months significantly increased antioxidant enzyme levels (SOD, CAT, and GPx) and improved blood pressure markers [85]. In addition, the same dose of rutin reduced levels of fasting blood glucose, insulin, HbA1c, triglycerides, total cholesterol, IL-6, and malondialdehyde. Rutin intake was also associated with increased brain-derived neurotrophic factor (BDNF), which is essential for neurogenesis, synaptic plasticity, and overall brain health [86]. Although there are several contradictory findings, various studies have found lower serum levels of BDNF in T2DM patients, which appears to be linked to insulin resistance and the duration of diabetes. Thus, BDNF might be involved in metabolic control and could play a role in the regulation of glucose metabolism and insulin sensitivity [87]. In a study by Sattanathan et al. [88], T2DM patients received 500 mg of rutin for 60 days. This supplementation lowered fasting blood glucose levels, and systolic and diastolic blood pressures, while increasing high-density lipoprotein (HDL) cholesterol levels. In general, catechins have been shown to inhibit glycogen synthesis, lipogenesis, and glucose oxidation in the liver and weaken glucose transporters in the intestine [25]. The intragastric administration of rutin from Tartary buckwheat reversed gut dysbiosis with increased favourable *Akkermansia*, *Roseburia*, and *Alisitipes* and reduced amount of *Escherichia* and *Mucispirillum* in diabetic mice. In addition, rutin decreased glucose and improved serum cholesterol, insulin, triglyceride concentrations, TNF-α, and IL-6 [89].

Epicatechin supplementation improved insulin resistance and fasting serum insulin levels in a clinical study but had no effect on fasting blood glucose levels [90]. On the other hand, epicatechin lowered hyperglycemia and improved insulin response to a glucose load, possibly by modulating pancreatic insulin production and secretion in rats [91]. Similarly, a decreased rate of weight gain, hyperglycemia, and hypertriglyceridemia was found after epicatechin administration in a rat model of high-fat diet (HFD)-induced obesity [92]. In addition, epicatechin alleviated the altered glucose uptake in HepG2 and NRK-52E cell lines [93,94], and demonstrated hydrolyzing enzyme inhibitor properties [82]. Kobayashi et al. [95] reported that epicatechin gallate is able to inhibit SGLT1. In HFD- and streptozotocin (STZ)-induced diabetic mice, epigallocatechin gallate improved glucose homeostasis and inhibited gluconeogenesis and lipogenesis in the liver [96]. Epicatechin, a dietary polyphenol, exerted a beneficial effect on gut microbiota with reduced lipopolysaccharide-producing bacteria, effectively protected glucose homeostasis, and elevated serum level of insulin in T2DM Goto-Kakizaki rats [97]. In a study by Li et al. [98], epigallocatechin gallate (50 and 100 mg/kg for 20 weeks) enhanced lipid profile, suppressed the expression of genes related to fatty acid synthesis, and increased the expression of genes associated with lipolysis and lipid oxidation in white adipose tissue of HFD mice. In addition, galloylated catechins showed higher efficiency than non-galloylated catechins in α-glucosidase and α-amylase inhibition [25]. Within the isoflavones group, a randomized placebo-controlled study by Villa et al. [99], including 50 postmenopausal women, revealed reduced fasting blood glucose and enhanced glucose tolerance and insulin sensitivity after genistein administration (54 mg/day for 24 weeks). In murine models, genistein (600 mg/kg for 4 weeks) significantly lowered levels of triglycerides and glucose [100]. Even a lower dose of genistein (250 mg/kg for 4 weeks) ameliorated hyperglycemia, glucose tolerance, and insulin levels in STZ-induced diabetic mice, which was accompanied by improved β-cell proliferation, survival, and mass [101]. In addition, genistein induced cyclic adenosine monophosphate/protein kinase A (cAMP/PKA) signalling and phosphorylation of extracellular signal-regulated kinase (ERK)1/2 in both INS1 cells and human islets [102]. Numerous other animal and in vitro studies have revealed beneficial effects of genistein on β-cell function [103]. Moreover, genistein is associated with gut microbiota changes and enhanced insulin resistance. A study on obese patients demonstrated that the consumption of genistein decreased insulin resistance and altered gut microbiota with increased Verrucomicrobia phylum [104]. In vitro and in vivo studies documented the preventive effect of daidzein against T2DM [105]. A case–control study by Nguyen et al. [106] including adult humans showed that dietary intake of daidzein correlated with a reduced risk of T2DM. Using non-obese diabetic mice, Choi et al. [107] demonstrated that daidzein supplementation (0.2 g/kg diet for 9 weeks) reduced fasting blood glucose and plasma insulin levels. Moreover, daidzein-treated mice have higher hepatic glucokinase and lower glucose-6-phosphatase and phosphoenolpyruvate carboxykinase activities, indicating that it has a positive impact on lowering neoglucogenesis and glycogenolysis. Other animal studies have confirmed improvements in glucose and lipid metabolism after daidzein administration (0.2 g/kg for 6 weeks; 0.1% for 4 weeks) [105,108,109]. Gut bacteria can convert daidzein into its metabolite equol [110]. In vivo experiments with Zucker diabetic fatty rats revealed that equol supplementation improved insulin secretion failure [111].

The meta-analysis by Guo et al. [112] investigated the association between dietary anthocyanins intake and the development of T2DM in 12,611 participants. According to their results, dietary anthocyanin consumption was linked to a 15% lower risk of T2DM. A double-blind, randomized, placebo-controlled study found that anthocyanin supplementation (320 mg/day for 24 weeks) along with standard treatment significantly lowered levels of triglycerides, LDL cholesterol, fasting plasma glucose, and oxidative stress markers while enhancing antioxidant capacity [113]. Another randomized controlled trial showed that administration of purified anthocyanins (320 mg/day for 12 weeks) positively affected glycemic control and lipid profiles in adults with prediabetes or early untreated diabetes. This study included 138 participants, and significantly decreased levels of HbA1c, LDL cholesterol, apolipoproteins A-1 (apo A1), and B (ApoB) were reported in the anthocyanin group [114]. Pomegranate is a potent antioxidant rich in flavonoids such as anthocyanins; its consumption improves glucose profile in adults [115,116]. In a mouse model, the supplementation with blueberry anthocyanins improved insulin sensitivity via modulated gut microbiota [117]. Combined treatment of metformin (MET) and anthocyanin increased beneficial bacteria with SCFA production and decreased the expression of PTP1B in diabetic mice [118]. PTP1B is a phosphatase regulating diabetes, obesity, and cancer-associated pathways [119,120]. Based on the results, anthocyanin may enhance the effectiveness of MET in the treatment of T2DM [118].

In an animal study, supplementation with scutellarein (50 mg/kg for 16 weeks) resulted in attenuation of obesity, insulin resistance, and oxidative stress [121]. In addition, the positive effect of tangeretin in relation to T2DM indicators was presented by Guo et al. [122] using primary hepatocytes and diabetic mice. Tangeretin (25 and 50 mg/kg for 30 days) ameliorated insulin sensitivity and improved glucose homeostasis in animal models. Moreover, in primary hepatocytes, tangeretin (10 and 20 μM for 48 h) suppressed the mitogen-activated protein kinase (MEK)-ERK1/2 pathway, which is known to regulate insulin sensitivity, and its inhibition improves insulin resistance. Additionally, the treatment of these cells led to the upregulation of insulin-stimulated Akt and glycogen synthase kinase 3 β (GSK3β) phosphorylation, increased glycogen content, and reduced glucose output. Tangeretin administration significantly changed gut microbiota with increased *Bacteroides* and *Lactobacillus* and improved insulin resistance and glucose intolerance in HFD mice [123].

Some flavonoids, like diosmetin, formononetin, and luteolin, demonstrated the ability to improve diabetic nephropathy. In STZ-induced diabetic mice, diosmetin administration (50 mg/kg for 5 days) significantly decreased fasting blood glucose level and ameliorated altered levels of oxidative stress parameters and inflammatory cytokines. Furthermore, diosmetin declined the expression of Akt and NF-κB [124]. The administration of diosmetin led to elevated serum insulin level while decreasing blood glucose concentration in diabetic mice. Moreover, diosmetin changed dysbiotic microbiota and elevated the level of *Corynebacterium glutamicum* [125]. The application of formononetin (10, 20 and 40 mg/kg for 16 weeks) showed a positive effect on hyperglycemia and insulin resistance in diabetic rats. Reduced levels of triglycerides and cholesterol were also noted [126]. The Qijian mixture, rich in formononetin, calycosin, and puerarin, changed gut microbiota and increased genera *Bacteroides*, *Senegalimassilia*, and *Clostridium sensu stricto 1* in diabetic mice. Gao et al. [127] confirmed the hypoglycemic effect of this traditional Chinese medicine in alleviating T2DM. Luteolin administration (10 and 20 mg/kg for 4 weeks) restored insulin resistance, dyslipidemia, hyperuricemia, and renal inflammatory cell infiltration in STZ-induced diabetic mice [128]. Developed porous starch microspheres loaded with luteolin alleviated injury in liver tissue caused by T2DM. The intervention altered gut microbiota with decreased unfavourable *Acetatifactor*, *Candidatus Arthromitus*, and *Turicibacter*. Further studies might be focused on the hypoglycemic potential of this natural flavonoid in T2DM [129]. The list of studies that investigated the relationships between flavonoids mentioned in this review and T2DM without gut microbiota composition is summarized in Table 1, and those that also looked at gut microbiota composition are listed in Table 2.

### 2.2. Phenolic Acids and T2DM

**Figure 2:**
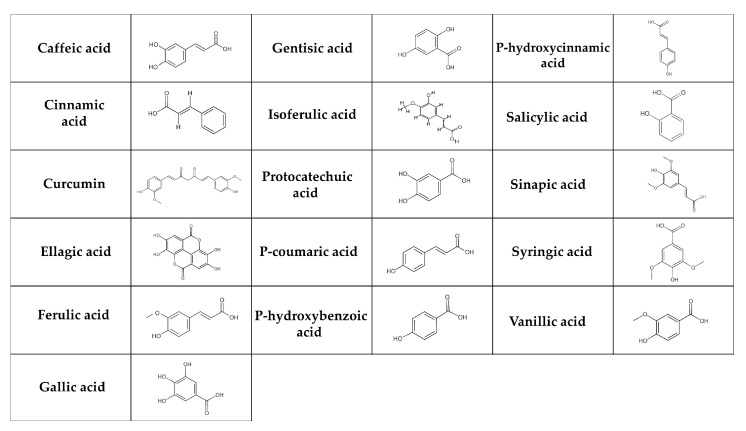
Chemical structure of phenolic acids. The simple phenolic acids can be classified as hydroxybenzoic (gentisic, salicylic, gallic, protocatechuic, ellagic, syringic, p-hydroxybenzoic, and vanillic acids) and hydroxycinnamic acids (caffeic, chlorogenic, cinnamic, isoferulic, p-coumaric, ferulic, and sinapic acids, curcumin).

Phenolic acids are the most basic polyphenols in terms of their chemical structure [130]. These are carboxylic acids, divided into hydroxybenzoic acids (e.g., gallic, p-hydroxybenzoic, salicylic, ellagic, gentisic, protocatechuic, syringic and vanillic acids) and hydroxycinnamic acids (e.g., cinnamic, p-coumaric, caffeic, ferulic, isoferulic, sinapic, and chlorogenic acids, curcumin) (Figure 2) [131,132]. The salicylic and gallic acids are synthesized from aromatic amino acids produced through the shikimate pathway [131] in chloroplast [133]. Other hydroxybenzoic and hydroxycinnamic acids are synthesized in the cytosol [134]. In general, phenolic acids can be found in cereals (e.g., barley, oats, rice, wheat), seeds (e.g., quinoa, chia seeds, flax seeds), fruits (e.g., grapes, pears), berries (e.g., blackberries, strawberries), vegetables (e.g., carrots, cabbage, spinach), nuts (e.g., walnuts), spices (e.g., cinnamon, vanilla pods), medicinal herbs (e.g., chicory), tea, cocoa, coffee, algae, and fungi [135,136,137]. They are broken down in multiple tissues and by the intestinal microbiota in the digestive tract. Since only about 10% of degraded phenolic acids is available in the small intestine, the majority is transported to the large intestine [138].

Hydroxybenzoic acid breaks down into hippuric acid and glucuronide. This process is highly saturating and affected by the availability of glycine. At high doses, it leads to the urea cycle, gluconeogenesis, fatty acid metabolism, and tricarboxylic acid cycle confiscating coenzyme A prior to its conjugation with glycine [139]. Ellagic acid is absorbed within one hour after consumption [140]. Its metabolite, urolithin B, is conjugated with glucuronic acid and excreted in the urine [141]. Most of the gallic acid is absorbed through the upper digestive tract and the rest through the stomach [142]. Additionally, following ingestion, salicylic acid is found in the blood and urine [143]. Hydroxycinnamic acids are degraded directly in the large intestine, absorbed through the barrier of the gastrointestinal tract, and subsequently enter the peripheral bloodstream [144]. According to El-Seedi et al. [145], cinnamic acid is found in plasma immediately after consumption. Some other phenolic acids, such as caffeic, p-coumaric, and ferulic acids are absorbed through the monocarboxylic acid transporters, across the intestinal epithelial cells [146]. P-coumaric acid is subsequently metabolized in the liver [145].

Although the biological effects of phenolic acids are still controversial, their positive impact on T2DM is undeniable [147]. Phenolic acids can improve the symptoms of T2DM by regulating hepatic glucose homeostasis and influencing carbohydrate metabolism pathways (by inhibiting α-glucosidase and α-amylase) [148,149]. Chlorogenic acid might increase insulin sensitivity and decrease inflammation [5]. A meta-analysis of 10 clinical studies investigated the effects of green coffee bean extract (GCE) supplementation, focusing on chlorogenic acid. A total of 363 participants were included with GCE dosages ranging from 100 to 1000 mg/day for 2 to 16 weeks. The summary from this meta-analysis revealed a significant reduction in fasting blood glucose levels (especially at doses of ≥400 mg/day), but insulin levels were not altered. Some subgroup analyses also highlighted significant improvements in insulin levels after GCE supplementation [150]. To track the effects of chlorogenic acid at a dosage of 1200 mg/day for 12 weeks, 30 subjects with impaired glucose tolerance (IGT) participated in another randomized, double-blind, placebo-controlled clinical trial. They were not taking any other drugs or medications during the trial. Chlorogenic acid administration significantly lowered levels of fasting blood glucose, LDL cholesterol, and triglycerides, and there was an increase in the Matsuda index, indicating improved insulin sensitivity [151]. In diabetic mice, chlorogenic acid administration elevated *Lactobacillus*, *Blautia*, and *Enterococcus* within the gut microbiota. The beneficial effect of this phenolic acid contributed to improved glucose tolerance [152].

There is insufficient evidence from clinical trials specifically monitoring the administration of extracted caffeic acid to patients with diabetes-related symptoms. However, numerous studies have demonstrated the effects of plant and animal products on T2DM, with caffeic acid often cited as a component. The review by Ganguly et al. [153] explored the impacts of caffeic acid on DM-related complications. It highlighted three clinical trials involving propolis as a source of caffeic acid and caffeic acid phenethyl ester. These studies showed that giving propolis to DM patients at doses of 300 and 400 mg for 2, 3, or 6 months increased the Matsuda index, 2-Deoxy-D-glucose (2-DG) uptake, and GPx levels while significantly lowering fasting blood glucose and HbA1c levels. The water extract derived from the leaves of *Lycium barbarum* L., rich in neochlorogenic acid, chlorogenic acid, caffeic acid, and rutin improved gut dysbiosis and reduced insulin resistance in diabetic rats [154]. Ellagic acid has been shown to exert hypoglycemic effects and is able to improve glycemic control. In a clinical trial, consumption of ellagic acid (180 mg/day for 8 weeks) significantly decreased levels of glucose, insulin, HbA1c, total cholesterol, triglycerides, LDL cholesterol, malondialdehyde, C-reactive protein, TNF-α, and IL-6. However, the values of total antioxidant capacity and activities of SOD and GPx were elevated [155]. In the research by Hosseini et al. [156], 24 overweight and obese adults received 500 mg of pomegranate extract with 40% ellagic acid twice a day for 1 month, and a substantial decrease in homeostatic model assessment for insulin resistance (HOMA-IR) was recorded. Kang et al. [157] documented numerous beneficial effects of ellagic acid against obesity-associated health problems by altering gut microbiota.

An umbrella meta-analysis included 22 studies with a total of 1600 participants and examined the impacts of curcumin supplementation on glycemic indices. The doses of curcumin ranged from 0.08 to 2 g/day, and the duration of the trials varied from 4 to 30 weeks. Curcumin significantly reduced levels of blood glucose, HbA1c, and insulin. Participants involved in this analysis had a variety of health problems, including T2DM, polycystic ovary syndrome, non-alcoholic fatty liver disease, and metabolic syndrome. Notably, many of the included studies focused on populations with insulin resistance, a condition associated with DM [158]. When compared to conventional medication, curcumin supplementation dramatically lowered fasting blood glucose and HbA1c levels in T2DM patients, according to another systematic review and meta-analysis. The meta-analysis included five trials with 349 participants and curcumin doses ranging from 80 to 2100 mg/day [159]. A controlled clinical trial by Hodaei et al. [160] included 53 participants with T2DM and discovered that receiving 1500 mg of curcumin daily for ten weeks considerably lowered fasting blood glucose levels. In animal and in vitro studies, the effect of other phenolic acids on T2DM has been examined. Huang et al. [161] documented that curcumin restored gut microbiota balance by decreasing *Enterobacterales* and Firmicutes and improved insulin resistance in diabetic rats.

Natural chicory extract (*Cichorium intybus*) rich in chicoric, chlorogenic, and caffeoylquinic acids enhanced glucose tolerance and reduced basal hyperglycemia in STZ-induced diabetic rats [162]. In HFD mice, chicoric acid also changed the gut microbiota by raising the ratio of Firmicutes to Bacteroidetes [163]. According to Peng et al. [164], chicoric acid enhanced glucose uptake in cell and murine models and increased Akt phosphorylation. HFD mice given 0.5% ferulic acid for 7 weeks showed significantly lower levels of glucose, glucose-6-phosphatase, and phosphoenolpyruvate carboxykinase activities, as well as reduced hyperlipidemia and oxidative stress. They also exhibited elevated insulin and glycogen concentrations [165,166]. Ferulic acid (10 mg/kg for 15 days) demonstrated antioxidant as well as antidiabetic effects and ameliorated liver, kidney, and pancreas damage in mice with alloxan-induced diabetes, presumably through NF-κB inhibition [167]. Ferulic acid (10 and 40 mg/kg for 3 weeks) was found to significantly lower blood glucose, urea, creatinine, glutamic pyruvic transaminases, glutamic oxaloacetate transaminases, and improve the lipid profile in STZ-induced diabetic rats [168]. According to Narasimhan et al. [169], ferulic acid has the ability to regulate the expression of the GLUT2 gene in the liver in a rat model of T2DM. Song et al. [170] determined the protective effect of ferulic acid against diabetic syndrome via modulated gut microbiota with reduced *Lactobacillus*, *Ruminococcus*, and promoted *Bacteroides*, *Blautia*, *Faecalibacterium*, *Parabacteroides* and *Phascolarctobacterium*.

Rats with alloxan-induced diabetes who were given syringic acid (50 mg/kg for 30 days) showed an increase in insulin and C-peptide levels and a decrease in plasma glucose levels [171]. In the study by Srinivasan et al. [172], syringic acid was administered intragastrically to diabetic rats (25, 50, and 100 mg/kg for 30 days). According to the findings, it significantly improved the levels of plasma glucose, HbA1c, insulin, and glycogen as well as the functional markers of the liver and kidneys. It also restored the activities of important enzymes involved in the carbohydrate metabolism. Yoon et al. [173] demonstrated that L6 skeletal muscle cells treated with 0, 12.5, 25, 50, and 100 μM of p-coumaric acid for 48 h were capable of modulating glucose and lipid metabolism through AMPK activation. Sinapic acid (5, 10, and 25 mg/kg for 3 days) exerted a hypoglycemic effect in STZ-induced diabetic rats [174]. Derebasi et al. [175] revealed a positive impact of p-coumaric acid on probiotic *Lactobacillus* spp. in vitro.

Cinnamic acid demonstrated antidiabetic activity by enhancing glucose tolerance in rats with STZ-induced diabetes (5 and 10 mg/kg for 14 h) and inducing insulin secretion in isolated islets. Cinnamic acid (10 mg/kg) produced an improvement that was similar to that of glibenclamide (5 mg/kg), a standard drug [176].

The antihyperglycemic, antioxidant, and antilipid peroxidative impacts of gallic acid (10 and 20 mg/kg for 21 days) were demonstrated by Punithavathi et al. [177], which protected the pancreas from diabetes induced by STZ in rats. The elevated insulin secretion after gallic acid treatment positively reversed the impaired carbohydrate metabolism by lowering gluconeogenesis and enhancing glycolysis, ultimately leading to a reduction in hyperglycemia. In another study, gallic acid (20 mg/kg for 30 days) decreased body weight gain, fasting blood glucose and insulin levels in diabetic rats. It also significantly improved the level of PPAR-γ expression, and enhanced glucose uptake through translocation and activation of GLUT4 in the PI3K/Akt signalling pathway [178]. Gallic acid isolated from *Terminalia bellerica* Roxb. (5, 10, 20 mg/kg for 28 days) reduced levels of plasma glucose, triglyceride, total cholesterol, LDL cholesterol, creatinine, and uric acid in STZ-induced diabetic rats. Conversely, it considerably raised the concentrations of insulin, total protein, C-peptide, glycogen, and albumin [179]. A lower level of gallic acid has been determined in patients with adult-onset type 1 diabetes. The authors observed that a decreased abundance of SCFA-producing bacteria correlated with reduced gallic acid levels [180]. Table 3 shows the list of phenolic acids included in this review in connection with T2DM.

### 2.3. Stilbenes and T2DM

**Figure 3:**
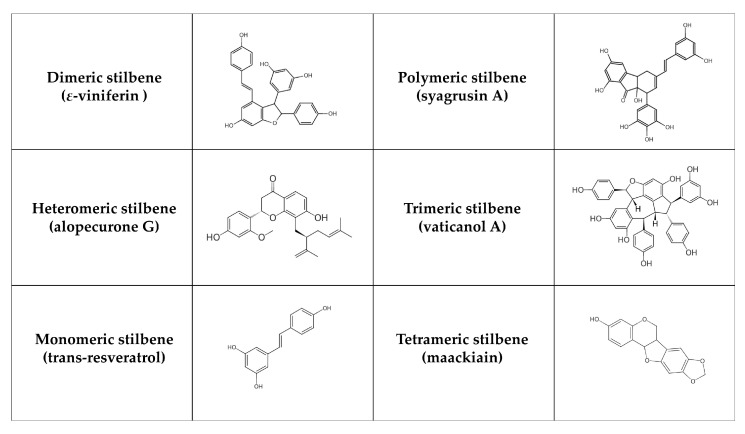
Chemical structure of selected stilbenes.

Stilbenes are formed in plants (e.g., *Vitaceae*, *Leguminaceae*, *Gnetaceae*, and *Dipterocarpaceae* families) as protective agents that protect against external stressors like UV radiation, infection, and pathogen attack [181,182]. Stilbenes can often be identified in grapes as well as in other plants and fruits such as rhubarb, banana, guava, pineapple, apple, peach, passion fruit, pears, and peanuts [183]. Structurally, their main characteristic is the presence of a 1,2-diphenylethylene nucleus [184]. According to their polymerization, monomeric and oligomeric stilbenes can be identified, where oligomeric stilbenes are formed of homogeneous or heterogeneous monomers of stilbene. In general, stilbenes can be divided into six groups [185]: monomeric (styrastilbene A; 13-hydroxykompasinol A; resveratrol; cajanonic acid A; hydrangeic acid; hypargystilbene B, D, E; lonchocarpene; rumexoid), dimeric (multiflorumiside J, K; polygonumnolide D; scirpusin C; *ε*-viniferin), trimeric (α-viniferin; passiflorinol B; vaticanol A, E, G), tetrameric (maackiain; paeonilactiflorol), polymeric (syagrusin A, B) and heteromeric (polyflavanolstilbene A) (Figure 3). Stilbenes exist in trans and cis isomeric forms, with trans-stilbene being the most common [182]. The metabolism of stilbenes is very rapid. Although 70% of resveratrol taken orally is absorbed, only 5 ng/mL is ultimately accessible, and its half-life in plasma is 9 h [186]. Subsequently, transformed compounds (in the large intestine) are further degraded or excreted by the liver [187]. These compounds are metabolized by uridine 5′-diphosphate glucuronide transferase, sulfotransferase, and catechol O-methyltransferase enzymes. In this phase, metabolism produces water-soluble inactive compounds, which can be easily eliminated [188]. The chemical structure of stilbenes affects the speed of their metabolism. For instance, the presence of a hydroxyl group in oxyresveratrol increases its susceptibility to glucuronidation and sulfation reactions, resulting in a poorer pharmacokinetic profile [189]. On the other hand, pinostilbene contains the dimethoxy group in cycle A instead of the hydroxyl group. Therefore, it is more resistant to metabolism [190]. Overall, the replacement of the hydroxyl group leads to an improvement in stilbene properties. Additionally, acetyl-trans-resveratrol, which contains acetyloxy groups, is more capable of penetrating the cell membrane. Trans-2,3,5,4′-tetrahydroxystilbene-2-O-glucoside, including hydrophilic groups, is more stable and soluble in water than resveratrol [191]. Higher oligomerization and intraperitoneal doses also decrease bioavailability. For example, ε-viniferin has a biological activity of more than 90% after intraperitoneal administration compared to 0.77% after oral administration [192].

Stilbenes may influence T2DM and its comorbidities. After six years of follow-up, an observational cohort study of 3430 non-diabetic participants showed that the highest tertile of stilbene intake reduced the risk of developing new-onset diabetes by 43% [74]. Considering individual stilbenes, various animal studies suggest that resveratrol can increase sirtuin 1 (SIRT1) expression, which stimulates PPAR-γ coactivator 1-α activity. The subsequent upregulation of GLUT4 and AMPK expression is linked to improved insulin sensitivity in peripheral tissues [193,194]. According to Peng Goh et al. [195], 10 patients with T2DM were treated with 3 g/day of resveratrol for 12 weeks. Resveratrol treatment regulated energy expenditure by upregulating AMPK and SIRT1 expression in skeletal muscle, suggesting that it may have favorable exercise-mimetic effects in T2DM patients. In another study, 19 individuals suffering from T2DM received resveratrol (10 mg/day for 4 weeks). This supplementation ameliorated insulin sensitivity, possibly due to less oxidative stress, which in turn led to more efficient insulin signalling through the Akt pathway [196]. The intervention with resveratrol increased the abundance of *Erysipelotrichaceae* and *Ileibacterium* in the gut microbiota, while also enhancing insulin sensitivity and glucose tolerance in T2DM mice. The authors [197] noted that, following resveratrol treatment, the microbiota composition closely resembled that of healthy control mice. In a clinical study by Bhatt et al. [198], resveratrol (250 mg/day for 3 months) significantly improved systolic blood pressure, HbA1c, total cholesterol, and total protein levels in 62 participants. However, no significant changes were observed in body weight, LDL and HDL cholesterols. Movahed et al. [199] examined the antihyperglycemic effect of resveratrol (40 and 500 mg/day for 6 months) in 192 patients with T2DM. According to their findings, these patients benefited greatly from resveratrol supplementation in conjunction with conventional antidiabetic treatment, including significant decreases in blood glucose, HbA1c, and insulin levels, mitigating insulin resistance, and HDL cholesterol levels. An important role of PTP1B and *α*-glucosidase in the pathophysiology and complications of T2DM has been revealed in vivo and in vitro [200]. It has been demonstrated that stilbenes serve as their potent inhibitors (e.g., styrastilbene A, paeonilactiflorol, 13-hydroxykompasinol A, scirpusin C, *ε*-viniferin, passiflorinol B, polyflavanostilbene A, polygonumnolide D, rumexoid, syagrusin A, B, vaticanol A, E, G), and therefore can have antidiabetic effects [201,202,203,204,205,206,207,208,209,210,211,212]. Cajanonic acid A has been reported to exert inhibitory activity on PPAR-γ and PTP1B. Its hypoglycemic properties in rats (15, 30 and 60 mg/kg for 24 h) were comparable to that of the approved antidiabetic drug rosiglitazone [213]. The relationship of hydrangeic acid to DM was observed by Zhang et al. [214]. Hydrangeic acid markedly elevated the amount of adiponectin in 3T3-L1 cells, 2-deoxyglucose uptake into cells, and GLUT4 translocation. Higher mRNA levels of adiponectin, GLUT4, and PPAR-γ were documented, while TNF-α mRNA expression was reduced. Furthermore, this acid (200 mg/kg/day for 2 weeks) significantly lowered levels of blood glucose, triglycerides, and free fatty acids in a mice model. The effect of two stilbenes, lonchocarpene and 3,5-dimethoxy-4′-O-prenyl-trans-stilbene (DPS), on α-glucosidase activity and postprandial hyperglycemia was investigated by Pereira et al. [205]. Both stilbenes inhibited α-glucosidase activity in vitro. Furthermore, DPS caused a significant reduction in hyperglycemia in a mice model, whereas lonchocarpene did not. Recently, the associations between cajanonic acid A, hydrangeic acid, lonchocarpene, and 3,5-dimethoxy-4′-O-prenyl-trans-stilbene with gut microbiota in T2DM or metabolic diseases have not been fully investigated. Table 4 provides the list of aforementioned stilbenes in relation to T2DM.

### 2.4. Tannins and T2DM

**Figure 4:**
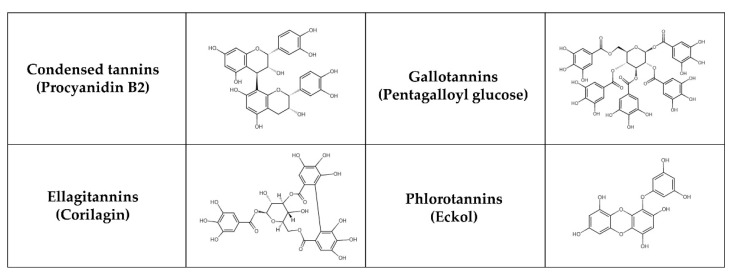
Chemical structure of selected tannins.

Tannins, natural phenolic compounds, are found in plants and can combine with a variety of minerals and macromolecules, including proteins, cellulose, and starch, to form a strong complex [215,216]. Different groups of tannins can be distinguished based on their chemical structure (Figure 4): hydrolysable tannins—HTs (e.g., gallotannins—GTs and ellagitannins—ETs), condensed tannins—CTs (syn. proanthocyanidins, non-hydrolysable tannins, e.g., procyanidins) and phlorotannins [217]. HTs with smaller molecules consist of sugar esters of polyphenolic carboxylic acids, e.g., gallic or ellagic acids. CTs may be derived from flavanols, such as catechin, flavan-3,4-diols or stilbenes. Polymerization results in more complicated structures [218]. Phlorotannins are present in brown seaweeds and are formed by polymerization of phloroglucinol units. Some classifications also recognize complex tannins (e.g., acutissimins A and B, camelliatannin A), composed of flavane-3-ol, the unit of CTs and HTs, which are linked by carbon-carbon bonds [219,220].

Tannins (except for phlorotannins) are most abundant in cocoa beans, tea, wines (especially red wine), fruits (e.g., apple, berries, plum, pomegranate), juices, nuts (e.g., cashew nuts, peanuts, walnuts), chocolate, legumes (e.g., chickpeas, cowpeas, lentils) and cereal grains (e.g., barley, sorghum) [221]. They can be metabolized in two phases. In the first metabolic phase (non-microbiota mediated), the biological effects of tannins are significantly influenced by their rate of decomposition and absorption. HTs are cleaved in the stomach or small intestine into monomers such as gallic or ellagic acids. Gallic acid from tea is rapidly absorbed and eliminated [222], while its derivatives are absorbed more slowly [142]. For ETs, the duration of intestinal absorption is between 0.5–3 h after oral administration in healthy subjects [140]. After absorption, free ellagic acid undergoes additional conjugation reactions with methyl, glucuronyl or sulfate groups, and these conjugates are detectable in urine and plasma [223]. CTs are absorbed intact from the small intestine, the rate of absorption being influenced by their stereochemistry and chemical structure [224]. After absorption, the aglycone forms of CTs undergo metabolism in the small intestine and later in the liver by glucuronide formation in the endoplasmic reticulum or by sulfonation and methylation in the cytosol [225]. The larger the CTs molecule and the more hydrophilic hydroxyl groups it contains, the lower the absorption rate [226]. In the second metabolic phase (microbiota-mediated), HTs are metabolized by various bacterial enzymes into gallic acid, pyrogallol, phlorogluciol and sometimes also into acetate and butyrate. GTs are later hydrolyzed and degraded by tannase to the hexahyroydiphenoyl. On the contrary, bacterial hydrolysis of ETs undergoes lactonization to produce ellagic acid [225]. Further, ellagic acid is converted into urolithin A and urolithin B, which are able to be easily absorbed [227]. These derivatives have been detected in plasma and urine [228]. CTs in their intact form as dimers, trimers and tetramers are metabolized during phase II in the intestine and liver [229]. Most CTs reach the large intestine intact, where the gut microbiota breaks them down into phenolic acids and phenylvalerolactones [230]. Dietary CTs shifted the gut microbiota in rats toward tannin-resistant bacterial taxa such as *Enterobacteriaceae* and *Bacteroides* [231]. Both HTs and CTs can support the growth and function of beneficial bacteria [232]. An extract of red maple leaves enriched with glucitol-core containing GTs modulated the gut microbiota and elevated the levels of *Prevotella* and *Eubacterium* in HFD mice. This supplementation led to decreased fat mass and body weight, improved insulin resistance, and reduced inflammation [233]. In a pilot clinical trial, the administration of mango increased GTs and SCFA production while altering bacterial taxa in lean participants, but not in obese individuals [234]. Additionally, the probiotic *Lactobacillus plantarum* in the gut microbiota can degrade GTs into gallic acid, facilitating its absorption [235].

Tannins have shown promising benefits for T2DM management in several animal and in vitro studies [236]. In rats with STZ-induced diabetes, the administration of tannins (100 and 200 mg/kg of tannin fraction for 30 days) effectively reduced elevated levels of blood glucose, total cholesterol, triglycerides, and LDL cholesterol and restored insulin and HDL cholesterol. Moreover, tannins significantly renewed the activity of antioxidant enzymes (SOD, CAT) and reduced GPx, thus renewing the organs’ antioxidant status to almost normal levels [237]. According to Shahidi and Danielski [5], favorable effects of GTs (e.g., monogalloyl hexoside) and ETs (e.g., ellagic acid hexoside) on reducing oxidative stress, insulin resistance, dyslipidemia, and inflammatory state have been demonstrated. ETs (e.g., lagerstroemin, flosin B) demonstrated strong effects in promoting insulin-like glucose uptake and reducing adipocyte differentiation in 3T3-L1 adipocytes [238]. According to Pinent et al. [239], grape seed procyanidins extracts (GSPE) substantially reduced hyperglycemia in STZ-induced diabetic rats and stimulated glucose uptake in insulin-sensitive cell lines, suggesting that they can exhibit insulinomimetic properties. Additionally, Montagun et al. [240] revealed that procyanidins phosphorylate p38 MAPK and p44/p42 much more than insulin. In insulin-sensitive tissues, they are able to modulate lipogenesis and glucose uptake, as well as ameliorate their oxidative/inflammatory status. Procyanidins also have the ability to modulate the level of active glucagon-like peptide-1 (GLP-1) [241]. Procyanidin B2 (PB2) has become more important due to its inhibitory activity on the formation of AGEs [242] and is one of the major components of GSPE. According to Yin et al. [243], PB2 from grape seeds (30 mg/kg for 10 weeks) alleviated the disturbances of hepatic lipid metabolism in diabetic mice. Body weights, triglycerides, total cholesterol, and free fatty acid levels were all significantly lower, but fasting blood glucose levels were not. In an in vitro study, grape seed PB2 (150 µg/mL for 48 h) reduced adipogenesis of 3T3-L1 cells by targeting PPAR-γ with a mechanism involving miR-483-5p [244]. By enhancing the abundance of *Lachnospiraceae* NK4A136 group, *Alloprevotella*, *Akkermansia*, and *Faecalibaculum*, peanut skin procyanidins modulate the gut microbiota in T2DM mice. The treatment also elevated levels of IL-10 while reducing lipopolysaccharide, IL-6, and myeloperoxidase. Additionally, this supplementation enhanced gut integrity by increasing the expression of tight junction proteins in the murine colon [245]. Phlorotannins have been shown to exert antidiabetic properties due to their acarbose-like activity, stimulation of glucose uptake, and protection of pancreatic β cells from oxidative stress [246,247]. A small double-blind, randomized, placebo-controlled study found that a commercially available blend of brown seaweed (*Ascophyllum nodosum* and *Fucus vesiculosus*) with known inhibitory action on α-amylase and α-glucosidase activities (InSea2^TM^) was associated with reduced insulin incremental area under the curve and enhanced insulin sensitivity [248]. In a rat model, the same product lowered postprandial blood glucose level and peak insulin secretion [249]. Phlorotannins from *Cystoseira compressa* demonstrated anti-diabetic effects by reducing glucose, total triglycerides, total cholesterol levels, while increasing antioxidant capacity and insulin concentration in STZ-induced diabetic rats [250]. Similarly, postprandial blood glucose levels were decreased after an administration of ezoishige (*Pelvetia babingtonii* de Toni) extract in rats [251]. The list of tannins included in this review in connection with T2DM is summarized in Table 5.

### 2.5. Lignans and T2DM

**Figure 5:**
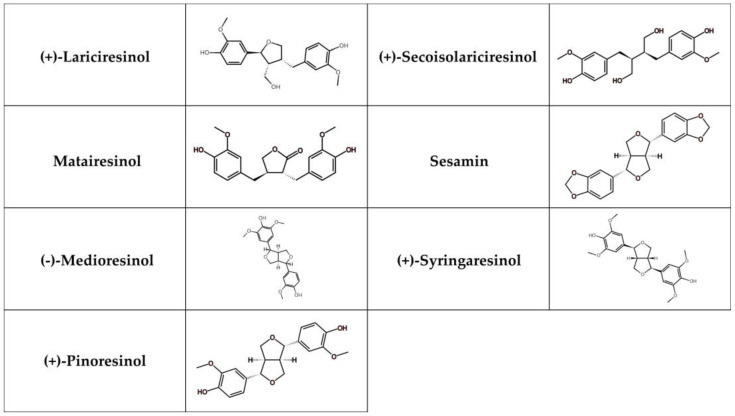
Chemical structure of selected lignans.

Lignans represent phenolic compounds derived from the biosynthesis of shikimic acid and are found in large quantities in plants [252]. Structurally, they form a part of a diverse family that includes lignans, neolignans, and oxyneolignans [253]. Lignans usually exhibit dimeric structures formed by a *β*, *β*’-linkage between two phenylpropane units. Neolignans, on the other hand, are compounds with an alternative linkage. Due to their chemical differences, lignans can be classified into several groups, such as secoisolariciresionol, pinoresinol, matairesinol, medioresinol, sesamin, syringaresinol, and lariciresinol (Figure 5) [254].

Lignans can be extracted from seeds (e.g., sesame, flaxseed), cereals (e.g., rye grains, buckwheat), vegetables (e.g., broccoli, cucumber), fruits (e.g., grapefruit, pear), beverages (e.g., red wine, coffee) and oils (e.g., olive oil, sesame seed oil) [255]. Numerous bioactivities of lignans result from the gut microbiota’s chemical transformation. Lignan glycosides are hydrolyzed to lignan aglycones and subsequently converted into enterolignans. These compounds have positive health effects and are more bioavailable than their precursors [256]. Sun et al. [257] proposed that enterolactone is linked to a decreased risk of T2DM in women. Lignan intake was related to higher serum enterolactone concentrations. The presence of *Coprococcus* sp. ART55/1, *Butyrivibrio crossotus*, *Faecalibacterium prausnitzii*, *Methanobrevibacter smithii*, and *Alistipes shahii* in the gut microbiota was associated with plasma enterolactone concentrations in participating men [258]. Furthermore, supplementation with a lignan-rich extract from *Cinnamomum camphora* leaves improved glucose and insulin tolerance while increasing the abundance of *Lactobacillus* in diabetic mice [259].

Several studies suggest that lignans intake may be associated with a lower risk of T2DM. A population-based prospective study conducted on 2882 men and 3665 women revealed that daily consumption of lignans was connected to decreased T2DM risk [260]. A cohort study by Wang et al. [261], involving 201,111 men and women from 3 large studies discovered that a lower risk of T2DM was associated with higher intakes of both total and individual lignans, with the exception of lariciresinol. On the other hand, in an observational cohort study of nondiabetic participants (n = 3430), no significant effect of consuming a lignan-rich diet on the development of T2DM was determined after 6.1 years of follow-up [74]. Several lignans have shown effects on various mechanisms related to T2DM. Pinoresinol was able to inhibit α-glucosidase [262], while syringaresinol reduced the levels of cyclooxygenase-2 (COX-2) and inducible nitric oxide synthase (iNOS) in microglia cells as well as the expression of inflammatory cytokines (TNF-α, IL-1β) [263]. Rats (carrageenan-induced model of inflammation) supplemented with sesamin (50 and 100 mg/kg for 2 days) showed lower levels of TNF-α, IL-1β, and IL-8 [264]. The treatment with secoisolariciresinol diglucoside, the glycosylated form of secoisolariciresionol, also decreased levels of IL-1β, IL-18, TNF-α, and NLR family pyrin domain containing 1 (NLRP1) in mice [265]. The aforementioned findings indicate that lignans may serve as potential therapeutic agents in inflammatory conditions, which are part of T2DM. Table 6 presents the list of lignans involved in this review related to T2DM.

## 3. The Interactions Between Plant-Derived Polyphenols and Antidiabetic Drugs

Since T2DM is a complex and complicated disease, treatment must be comprehensive. This complexity makes it impossible to control the disease and its risk factors with a single management approach [266]. In general, biguanides (e.g., MET), thiazolidinediones (e.g., rosiglitazone), insulin secretagogues (sulphonylureas such as glimepiride, meglitinides such as repaglinide), α-glucosidase inhibitors (e.g., acarbose), incretin-based therapies (GLP-1 agonists such as dulaglutide, dipeptidyl peptidase 4 (DPP4) inhibitors such as sitagliptin), and SGLT2 inhibitors (e.g., canagliflozin) belong to the common oral antidiabetic medications for T2DM [2,10]. However, the long-term application of these drugs can lead to adverse side effects, which emphasizes the need for new combination therapies aimed at the complex nature of the disease while minimizing toxicity [266]. For centuries, people around the world have been using traditional plant-based medicines [267]. Many T2DM patients supplement their conventional treatments with herbal medicines, which may influence the effectiveness of the treatment. Plant-drug interactions allow us to reveal how bioactive compounds in plants affect the pharmacokinetics (PK) and pharmacodynamics (PD) of synthetic drugs. PK involves drug absorption, distribution, metabolism, and excretion, primarily through cytochrome P450 (CYP450) enzymes [268,269]. Overall, three types of PD interactions/effects can be distinguished: synergistic, antagonistic, and additive [266,270]. Polyphenols in herbal medicines can interact and influence their bioactive properties. For example, quercetin and rutin from *Hippophae rhamnoides* L. may exhibit different PK interactions. In the polyherbal formulation, the bioavailability of quercetin was reduced, while that of rutin was increased, compared to co-administration [271]. However, studying these interactions is challenging because existing methods often oversimplify mixtures by focusing on individual compounds rather than the synergistic effects of whole plant extracts [272].

MET is the most common antidiabetic drug used in the treatment of T2DM patients. Multiple in vivo and in vitro studies have documented that the combination of different plant products (e.g., ferulic, p-coumaric, chlorogenic, caffeic, gallic acids, malvidin, cyanidin-3-arabinoside, anthocyanins, quercetin, kaempferol, apigenin, epigallocatechin gallate, avicularin) with MET has synergistic effects (Table 7) [118,168,273,274,275,276]. However, interactions between MET and certain herbal products can reduce its effectiveness by affecting drug transport mechanisms [277,278]. MET is taken up by organic cation transporter 1 (OCT1) for delivery to the liver and eliminated by multidrug and toxin extrusion protein 1 (MATE1) for renal excretion [279]. While combining MET with herbal products may enhance the benefits of treatment, it is essential to carefully assess potential negative interactions to optimize therapeutic outcomes. In this context, Knop et al. [277] revealed inhibitory effects of green tea and epigallocatechin gallate (EGCG) on multiple drug transporters in vitro. As a result, MET uptake mediated by OCT1 and MATE1 was significantly reduced in the presence of green tea and EGCG, suggesting an antagonistic interaction. However, in vivo combination therapy with MET and EGCG ultimately reduced fasting blood glucose in diabetic rats [280]. According to Haque et al. [278], *Abelmoschus esculentus* (L.) extract showed an agonist effect against the synergistic antidiabetic activity of MET and acarbose (ACR) when co-administered in glucose-induced hyperglycemic mice. Several studies indicate a synergistic effect of thiazolidinediones (TZDs) or PPAR-γ agonists, in combination with plant polyphenols. For example, TZDs combined with hydroxycinnamic derivatives significantly increased 2-DG levels [276], and TZDs combined with ferulic acid improved blood glucose and lipid profiles [168]. Pioglitazone with resveratrol also reduced fasting blood glucose levels and inflammation in diabetic rats [281]. However, naringenin, a citrus flavonoid, attenuated the hypoglycemic impacts of pioglitazone in obese diabetic mice, indicating weak partial antagonistic activity [282]. Several studies have demonstrated that sulfonylureas’ antidiabetic effects are lessened when combined with herbal products [283]. Co-administration of *Azadirachta indica* extract with glibenclamide or glimepiridine induced an antagonistic interaction, which was manifested by raised blood glucose levels [284]. Similarly, *Annona cherimola* extract did not show a decrease in blood glucose levels after co-administration with glibenclamide in diabetic mice, suggesting a possible antagonistic impact [285]. Gallic acid potentiated the impacts of ACR, an α-glucosidase inhibitor, in diabetic rats [275]. Rutin from *Annona cherimola* also showed positive effects when combined with ACR in diabetic mice [285]. Synergistic inhibition of α-glucosidase activity was observed when quercetin, baicalein, or luteolin were combined with ACR, while antagonistic inhibition was determined when (+)-catechin was combined with ACR [286]. Resveratrol improved the PK of DPP4 inhibitors (alogliptin and saxagliptin) in rats [287]. *Eugenia jambolana* extract combined with sitagliptin lowered fasting blood glucose levels and ameliorated lipid profile in diabetic rats [288]. The study by Valdes et al. [285] revealed that *Cherimoya annona* extract and rutin with canagliflozin (an SGLT2 inhibitor) significantly reduced blood glucose levels and improved lipid indicators in diabetic mice. The interactions of antidiabetic drugs with various plants and polyphenols obtained from them are summarized in Table 7.

## 4. Conclusions

**Figure 6:**
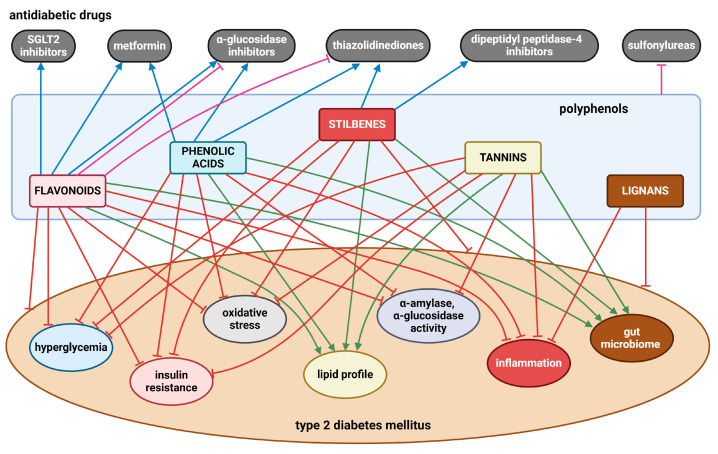
The effects of polyphenols on T2DM and its obvious clinical symptoms, as well as the interactions between polyphenols and antidiabetic drugs. Blunt red arrows indicate an inhibitory effect; sharp green arrows designate a stimulatory effect or improvement. Blue arrows show a synergistic effect, while blunt purple arrows indicate an antagonistic effect. Created with BioRender.com (accessed on 4 December 2024).

Numerous in vivo and in vitro investigations show that a diet high in polyphenols is linked to multiple health advantages. In T2DM, various flavonoids (e.g., quercetin, kaempferol, rutin, epicatechin, genistein, daidzein, anthocyanins), phenolic acids (e.g., chlorogenic, caffeic, ellagic, gallic acids, curcumin), stilbenes (e.g., resveratrol), tannins (e.g., procyanidin B2, seaweed phlorotannins), and lignans (e.g., pinoresinol) have been demonstrated to lower hyperglycemia, enhance insulin sensitivity and improve insulin secretion, scavenge ROS, and reduce chronic inflammation. In addition, mounting evidence highlights the role of polyphenols in modulating gut microbiota, leading to the abundance of beneficial microbial populations such as *Bifidobacteria* and *Lactobacillus*. However, findings remain inconsistent, with some studies reporting conflicting results. Several limitations hinder definitive conclusions, including variability in study design, differences in polyphenol bioavailability, structural variations among polyphenols that affect their metabolism, and significant individual heterogeneity in gut microbiota composition. Figure 6 presents the most significant findings related to this issue.

Importantly, dietary polyphenols also have the ability to mitigate serious secondary complications of T2DM, including cardiovascular disorders, diabetic neuropathy, nephropathy and retinopathy, diabetic foot, osteoporosis, liver damage, susceptibility to infections and some cancers. Since food generally contains several types of polyphenols in varying amounts, it is necessary to point out their mutual interactions. Polyphenols from guava tea, coffee, cocoa, olive oil, propolis, red wine, dark chocolate, blueberries, and grape seeds have been found to exert antidiabetic effects in patients with T2DM due to enhanced glucose metabolism, reduced insulin resistance, HbA1c, and improved vascular function. Based on available clinical studies, however, it seems that several individual polyphenols, such as quercetin, kaempferol, epicatechin, anthocyanins, curcumin, and resveratrol, have also demonstrated antidiabetic activity by reducing hyperglycemia, dyslipidemia, and alleviating insulin resistance.

The interactions between polyphenols and conventional antidiabetic drugs offer a promising strategy in the management and treatment of T2DM, especially when the disease is in an advanced stage, by lowering hyperglycemia, improving lipid profiles, and reducing insulin resistance. Although there is generally a promising potential for synergistic effects of polyphenols with antidiabetic drugs, the risk of antagonistic interactions that may impair drug efficacy (Figure 6) underlines the need for careful evaluation. In this direction, more research is required to clarify these mutual interactions with the aim of exploiting the knowledge in clinical applications. Several animal model studies and clinical trials published to date are often limited by small sample sizes, short experimental durations, and the diversity of parameters analyzed. An important limitation of studies analyzing different polyphenolic extracts is insufficient characterization of the experimental material or inadequate phytochemical analysis of the extracts as recommended by the best practice guidelines. Future research should therefore aim to address these shortcomings and include large, controlled clinical studies with diverse populations, taking into account individual variability and confounding factors such as diet and lifestyle.

However, based on the published data, it can be concluded that dietary polyphenol intake can alleviate the overt clinical symptoms of T2DM, as well as its secondary complications. This suggests that they undoubtedly play a protective role in the management and treatment of T2DM.

## Figures and Tables

**Table 1:** List of flavonoids in relation to T2DM without investigating the composition of gut microbiota.

Flavonoids	Research Models	Applied Treatment	Significant Observations	References
Flavonoid-rich diet	Clinical trial, cohort analysis: 3430 non-diabetic participants (1314 men and 2116 women; 65.2 ± 6.3 and 67.5 ± 5.6 years old)	3 groups according to the source of polyphenols: a, Mediterranean diet supplemented with extra-virgin olive oil; b, Mediterranean diet supplemented with nuts; c, Low-fat control diet. Duration: 5.5 years	↓ risk of developing T2DM in elderly individuals with higher polyphenol intake	[74]
Quercetin	Clinical trial: 24 diabetic patients (n = 12 in each group)	400 mg single oral dose	↓ postprandial hyperglycemia	[75]
Quercetin	Clinical trial: 92 male smokers (30–60 years old) (n = 49 quercetin group, n = 43 placebo group)	100 mg/day for 10 weeks	↓ glucose ↓ total cholesterol ↓ LDL cholesterol ↑ HDL cholesterol	[76]
Quercetin	In vivo: ICR mice (n = 5 in each group) In vitro: L6 myotubes studies	In vivo: 10, 100, 1000 mg/kg once for the observation of GLUT4 translocation In vitro: 0.1 nM, 1 nM, 10 nM, 1 µM and 10 µM for 15 min	↑ insulin signalling molecules ↑ activation of IRS1/PI3K/Akt pathway	[80]
Kaempferol	In vivo: 72 HDF male mice (n = 9 in each group) In vitro: HepG2 cells, primary mice hepatocytes	In vivo: 50 mg/kg/day for 6 weeks In vitro: 0.1, 1, 10, and 50 µM for 5 h	↓ glucose ↑ insulin sensitivity ↓ gluconeogenesis by inhibiting pyruvate carboxylase and glucose-6-phosphatase	[83]
Kaempferol	Clinical trial: 50 diabetic patients (n = 25 in each group)	1 g/day for 3 months	↑ GPx, CAT, SOD ↑ improved blood pressure markers	[85]
Rutin	Clinical trial: 50 diabetic patients (n = 25 in each group)	500 mg/day for 3 months	↓ FBG ↓ HbA1c ↓ insulin levels ↓ triglycerides ↓ cholesterol ↓ IL-6 ↓ MDA ↑ total antioxidant levels ↑ BDNF	[86]
Rutin	Clinical trial: 30 diabetic patients (45–50 years old)	500 mg/day for 60 days	↓ FBG ↓ systolic and diastolic blood pressure ↑ HDL cholesterol	[88]
Epicatechin	Clinical trial: 37 non-diabetic participants (40–80 years old)	100 mg/day for 4 weeks	↑ insulin ↓ insulin resistance	[90]
Epicatechin	In vivo: Wistar rats with glucose intolerance (n = 8 in each group)	1 mg/kg for 15 days	↑ glucose tolerance ↑ insulin secretion ↑ fasting insulin concentration	[91]
Epicatechin	In vivo: Wistar rats with HFD-induced obesity (n = 10 in each group)	1 mg/kg for 2 weeks	↓ weight gain ↓ glucose ↓ triglycerides	[92]
Epicatechin	In vitro: human HepG2 cells treated with high glucose and pre-treated with epicatechin	10 µM for 24 h	↓ insulin and IRS-1 levels ↓ inactivation of PI3K/Akt pathway	[93]
Epicatechin	In vitro: rat NRK-52E cells treated with high glucose and pre-treated with epicatechin	10 μM for 2 h	↓ insulin and IRS-1 levels ↓ inactivation of PI3K/Akt pathway	[94]
Epicatechin gallate	In vitro: brush-border membrane vesicles (BBMVs)	1 mM	↓ glucose uptake by SGLT1	[95]
Epigallocatechin gallate	In vivo: STZ- and HFD-induced mice with T2DM (n = 5 in each group)	300 mg/kg body weight for 3 weeks	↓ α-amylase ↓ α-glucosidase ↓ postprandial blood glucose ↓ insulin resistance ↓ expression of lipogenic genes	[96]
Epigallocatechin gallate	In vitro: HFD-mice (n = 8 in each group)	50 and 100 mg/kg for 20 weeks	↓ triglycerides ↓ total cholesterol ↓ LDL cholesterol ↑ HDL cholesterol ↓ expression genes involved in lipid metabolism	[98]
Genistein	Clinical trial: 50 postmenopausal women (53.91 ± 3.94 years old) (n = 30 genistein group, n = 20 control group)	54 mg/day for 24 weeks	↓ FBG ↓ basal insulin levels ↑ HDL cholesterol	[99]
Genistein	In vivo: ob/ob mice (n = 6 in each group)	600 mg/kg for 4 weeks	↓ triglycerides ↓ glucose	[100]
Genistein	In vivo: STZ-induced diabetic male C57BL/6 mice (n = 8 in each group)	250 mg/kg for 4 weeks	↑ glucose tolerance ↑ β-cell proliferation	[101]
Genistein	In vitro: INS1 cells and human islets	1 μM for 24 h	↑ cAMP/PKA signalling ↑ phosphorylation of ERK1/2	[102]
Daidzein	Clinical trial: 599 diabetic patients (40–65 years old)	≤5.2 mg/day, 5.3–9.8 mg/day, >9.8 mg/day for 2 years	↓ risk of T2DM	[106]
Daidzein	In vivo: Non-obese diabetic mice (n = 10 in each group)	0.2 g/kg for 9 weeks	↓ FBG ↓ plasma insulin ↓ glucose-6-phosphatase ↓ phosphoenolpyruvate carboxykinase ↑ hepatic glucokinase	[108]
Daidzein	In vivo: db/db mice (n = 10 in each group)	0.1% for 4 weeks	↓ FBG ↓ serum total cholesterol levels	[105]
Daidzein	In vivo: C57BL (db/db) mice (n = 10 in each group)	0.2 g/kg for 6 weeks	↑ insulin/glucagon ratio	[108]
Anthocyanin-rich diet and berries	Meta-analysis: 3 cohort studies: 200,894 non-diabetic participants and 12,611 diabetic patients 5 cohort studies: 194,019 non-diabetic participants and 13,013 diabetic patients	7.5 mg/day for dietary anthocyanin 17 g/day for berry intake	15% reduction in the risk of developing T2DM	[112]
Anthocyanins	Clinical trial: 58 diabetic patients (56–67 years old)	320 mg/day for 24 weeks	↓ triglycerides ↓ LDL cholesterol ↓ FBG ↓ ROS ↑ antioxidant capacity	[113]
Anthocyanins	Clinical trial: 160 prediabetes participants (40–75 years old)	320 mg/day for 12 weeks	↓ HbA1c ↓ LDL cholesterol ↓ apo A1 ↓ apo B	[114]
Scutellarein	In vivo: HFD-fed C57BL/6 J male mice (n = 12 in each group)	50 mg/kg for 16 weeks	↓ obesity ↓ insulin resistance ↓ oxidative stress	[121]
Tangeretin	In vitro: Primary hepatocytes	10 and 20 μM for 48 h	↑ Akt and GSK3β phosphorylation ↑ glycogen ↓ glucose output ↓ MEK-ERK1/2 pathway	[122]
Tangeretin	In vivo: C57BL/6J mice (db/db) (n = 6 in each group)	25 and 50 mg/kg for 30 days	↑ insulin sensitivity ↑ glucose homeostasis	[122]
Diosmetin	In vivo: STZ-induced diabetic mice	50 mg/kg for 5 days	↓ FBG ↓ ROS ↓ inflammatory cytokines ↓ Akt and NF-κB	[124]
Formononetin	In vivo: STZ-induced diabetic rats	10, 20 and 40 mg/kg for 16 weeks	↓ insulin resistance ↓ triglycerides ↓ cholesterol	[126]
Luteolin	In vivo: STZ-induced diabetic mice (n = 8 in each group)	10 and 20 mg/kg for 4 weeks	↓ insulin resistance ↓ dyslipidemia ↓ hyperuricemia ↓ renal inflammatory cell infiltration	[128]

Abbreviations: ↑—increased; ↓—decreased; Akt—protein kinase B; apo A1—apolipoprotein A-1; apo B—apolipoprotein B; BDNF—brain-derived neurotrophic factor; cAMP/PKA—cyclic adenosine monophosphate/protein kinase A; CAT—catalase; ERK1—extracellular signal-regulated kinase; FBG—fasting blood glucose; GLUT4—glucose transporter type 4; GPx—glutathione peroxidase; GSK3β—glycogen synthase kinase 3 β; HbA1c—glycated hemoglobin; HFD—high-fat diet; IL-6—interleukin 6; IRS—insulin receptor substrate; LDL—low-density lipoprotein; HDL—high-density lipoprotein; MDA—malondialdehyde; MEK—mitogen-activated protein kinase; NF-κB—nuclear factor kappa B; PI3K—phosphatidylinositol-3-kinase; ROS—reactive oxygen species; SGLT1—sodium-glucose linked transporter 1; SOD—superoxide dismutase; STZ—streptozotocin; T2DM—type 2 diabetes mellitus.

**Table 2:** List of studies characterizing gut microbiota alterations after flavonoid intervention in T2DM.

Flavonoids	Research Models	Applied Therapy	Significant Observations	Microbiota Alterations	References
Quercetin	In vivo: db/db mice (n = 10 in each group)	0.1% and 0.2% for 10 weeks	↓ body weight ↓ serum insulin level	↑ *Lachnospiraceae bacterium 28*-4 ↓ *Bacteroides*, Proteobacteria, *Escherichia-Shigella*, and *Escherichia coli*	[78]
Rutin	In vivo: C57BL/6 J mice with HFD/STZ induced diabetes (n = 5 in each group)	100 and 200 mg/kg for 4 weeks	↑ body weight ↑ HDL cholesterol ↓ glucose, insulin, ↓ total cholesterol ↓ triglyceride, ↓ LDL cholesterol ↓ TNF-α and IL-6	↑ *Alistipes*, *Akkermansia*, and *Roseburia* ↓ *Escherichia* and *Mucispirillum*	[89]
Epicatechin	In vivo: Goto–Kakizaki male rats (n = 7 in each group)	40 and 80 mg/kg/day for 4 weeks	↑ glucose homeostasis ↓ oxidative stress ↓ liver damage ↑ insulin	↓ lipopolysaccharide-producing bacteria	[97]
Genistein	Clinical trial: 45 obese participants (20–60 years old)	50 mg/day for 2 months	↓ insulin ↓ insulin resistance	↑ Verrucomicrobia	[104]
Anthocyanins	In vivo: C57BL/6 mice with high-fat, high-sucrose diet (n = 13–14 in each group)	Whole blueberry powder (BB) 160 mg Anthocyanins (ANT) 17 mg Proanthocyanidin (PAC) 1 mg for 12 weeks	↑ glucose homeostasis (ANT and PAC) ↑ insulin sensitivity (ANT and PAC)	↑ *Muribaculum intestinale* (PAC) ↓ *Lachnospiraceae bacterium Choco86*, *Ruminococcus torques*, *Blautia hansenii*, and *Blautia* sp. N6H1-15 (ANT)	[117]
Tangeretin	In vivo: C57BL/6 mice with HFD (n = 9–12 per group)	100 mg/kg for 12 weeks	↑ glucose tolerance ↓ insulin resistance ↓ systemic chronic inflammation	↑ *Bacteroides* and *Lactobacillus* ↓ Firmicutes/Bacteroidetes ratio	[123]
Diosmetin	In vivo: KKay diabetic mice (n = 6 in each group)	20 and 60 mg/kg for 4 weeks	↓ FBG ↓ triglycerides, ↓ total cholesterol ↓ LDL cholesterol ↑ insulin	↑ *Corynebacterium glutamicum* ↓ Firmicutes/Bacteroidetes ratio	[125]
Qijian mixture containing formononetin, calycosin, puerarin	In vivo: KKay diabetic mice (n = 6 in each group)	1795 and 5385 g/kg/day for 8 weeks	↓ hyperglycemia	↑ *Bacteroides*, *Senegalimassilia*, and *Clostridium sensu stricto*	[127]
Luteolin	In vivo: STZ- and HFD-induced mice with T2DM (n = 10 in each group)	50 and 100 mg/kg porous starch with luteolin for 4 weeks	↓ FBG ↓ triglycerides, ↓ total cholesterol ↓ LDL cholesterol ↓ IL-6 ↑ IL-10	↓ Firmicutes/Bacteroidetes ratio with harmful members including *Acetatifactor*, *Candidatus-Arthromitus*, and *Turicibacter*	[129]

Abbreviations: ↑—increased; ↓—decreased; HFD—high-fat diet; T2DM—type 2 diabetes mellitus, FBG—fasting blood glucose; IL—interleukin, LDL—low-density lipoprotein.

**Table 3:** List of phenolic acids in connection with T2DM.

Phenolic Acids	Research Models	Applied Treatment	Significant Observations	References
Chlorogenic acid	Meta-analysis of 10 studies: 363 non-diabetic participants (>18 years old)	Green coffee bean extract 100 to 1000 mg/day for 2 to 16 weeks	↓ FBG ↓ insulin levels ↑ Matsuda index	[150]
Chlorogenic acid	Clinical trial: 30 patients with IGT (30–60 years old) (n = 15 in each group)	400 mg 3x/day for 12 weeks	↓ FBG ↓ LDL cholesterol ↓ triglycerides ↑ insulin sensitivity	[151]
Caffeic acid	Clinical trial: 93 diabetic patients	Propolis 300 and 400 mg for 2, 3, and 6 months	↓ FBG ↓ HbA1c ↑ Matsuda index ↑ 2-DG uptake ↑ GPx	[153]
Ellagic acid	Clinical trial: 44 non-diabetic participants (24–55 years old) (n = 22 in each group)	180 mg/day for 8 weeks	↓ glucose, insulin, HbA1c, MDA, total cholesterol, triglycerides, LDL cholesterol, C-reactive protein, TNF-α, IL-6 ↑ total antioxidant capacity, SOD, GPx	[155]
Ellagic acid	Clinical trial: 48 overweight and obese participants (30–60 years old) (n = 24 in each group)	Pomegranate extract with 40% ellagic acid 500 mg twice a day for 1 month	↓ HOMA-IR	[156]
Curcumin	Meta-analysis of 22 studies: 1600 non-diabetic participants (>18 years old)	0.08 to 2 g/day for 4 to 30 weeks	↓ FBG ↓ HbA1c ↓ insulin	[158]
Curcumin	Clinical trial: 349 diabetic patients	80 to 2100 mg/day for 4 to 12 weeks	↓ FBG ↓ HbA1c	[159]
Curcumin	Clinical trial: 53 diabetic patients (40–70 years)	1500 mg/day for 10 weeks	↓ FBG	[160]
Synthetic chicoric (70%) and chlorogenic (30%) acids mixture	In vivo: STZ-induced diabetic rats (n = 4 in each group)	15 mg/kg for 7 days	↑ glucose tolerance	[162]
Chicoric acid	In vivo: STZ-induced diabetic rats (n = 4 in each group)	15 mg/kg for 7 days	↑ glucose tolerance ↓ basal hyperglycemia	[162]
Chicoric acid	In vivo: C57BL/6J mice (n = 6–8 in each group)	5 mg/kg for 5 days	↑ glucose uptake ↑ Akt phosphorylation	[164]
Chicoric acid	In vitro: C2C12 myotubes	12.5, 25, and 50 µM for 24 h	↑ glucose uptake ↑ Akt phosphorylation	[164]
Ferulic acid	In vivo: HFD C57BL/6N mice (n = 4 in each group)	0.5% for 7 weeks	↓ glucose ↓ glucose-6-phosphatase ↓phosphoenol-pyruvate carboxykinase ↑ glycogen and insulin ↓ HFD-induced hyperlipidemia and oxidative stress	[165,166]
Ferulic acid	In vivo: Wistar albino mice (n = 6 in each group)	10 mg/kg for 15 days	↓ lipid peroxidation ↓ antioxidants ↓ NF-κB	[167]
Ferulic acid	In vivo: STZ-induced diabetic rats (n = 5 in each group)	10 and 40 mg/kg for 3 weeks	↓ glucose, lipid profile, urea, creatinine, glutamic pyruvic transaminases and glutamic oxaloacetate transaminases	[168]
Ferulic acid	In vivo: HFD Wistar rats (n = 6 in each group)	50 mg/kg for 30 days	↓ GLUT2	[169]
Syringic acid	In vivo: Alloxan-induced diabetic rats (n = 6 in each group)	50 mg/kg for 30 days	↓ glucose ↑ insulin ↑ C-peptide levels	[171]
Syringic acid	In vivo: Alloxan-induced diabetic rats (n = 6 in each group)	50 mg/kg for 30 days (perorally) 25, 50 and 100 mg/kg for 30 days (intragastrically)	↑ insulin ↑ glycogen ↓ HbA1c	[172]
p-Coumaric acid	In vitro: L6 rat skeletal muscle cells	0, 12.5, 25, 50 and 100 μM for 48 h	↑ AMPK	[173]
Sinapic acid	In vivo: STZ-induced diabetic rats (n = 8 in each group)	5, 10, and 25 mg/kg for 3 days	↓ glucose ↑ glucose uptake	[119]
Cinnamic acid	In vivo: STZ-induced diabetic rats (n = 6 in each group)	5 and 10 mg/kg	↑ glucose tolerance ↑ insulin secretion	[176]
Gallic acid	In vivo: STZ-induced diabetic rats (n = 8 in each group)	10 and 20 mg/kg for 21 days	↓ glucose ↓ HbA1c ↓ glucose-6-phosphatase ↑ insulin ↑ hepatic hexokinase	[177]
Gallic acid	In vivo: Male Wistar rats (n = 6 in each group)	20 mg/kg for 30 days	↓ body weight gain ↓ FBG, PPAR-γ ↓ insulin ↑ glucose uptake	[178]
Gallic acid	In vivo: STZ-induced diabetic rats (n = 7 in each group)	5, 10, 20 mg/kg for 28 days	↓ glucose ↓ triglyceride ↓ total cholesterol ↓ LDL cholesterol ↑ insulin, albumin ↑ C-peptide, glycogen	[179]

Abbreviations: ↑—increased; ↓—decreased; 2-DG—2-Deoxy-D-glucose; Akt—protein kinase B; AMPK—AMP-activated protein kinase; FBG—fasting blood glucose; GLUT2—glucose transporter type 2; GPx—glutathione peroxidase; HbA1c—glycated hemoglobin; HFD—high-fat diet; HOMA-IR—homeostatic model assessment for insulin resistance; IGT—impaired glucose tolerance; IL-6—interleukin 6; LDL—low-density lipoprotein; MDA—malondialdehyde; NF-κB—nuclear factor kappa B; PPAR-γ—peroxisome proliferator-activated receptor-gamma; SOD—superoxide dismutase; STZ—streptozotocin; TNF-α—tumor necrosis factor alpha.

**Table 4:** List of stilbenes in relation to T2DM.

Stilbenes	Research Models	Applied Treatment	Significant Observations	References
Stilbene-rich diet	Clinical trial: 3430 non-diabetic participants (1314 men and 2116 women)	0.04 (0–0.17) to 3.89 (2.77–6.95) mg/day	↓ risk of T2DM	[74]
Resveratrol	Clinical trial: 10 diabetic patients (40–69 years old)	3 g/day for 12 weeks	↑ AMPK and SIRT1	[195]
Resveratrol	Clinical trial: 19 diabetic patients (>18 years old)	10 mg/day for 4 weeks	↑ insulin sensitivity ↓ oxidative stress	[196]
Resveratrol	Clinical trial: 62 diabetic patients (30–70 years old)	250 mg/day for 3 months	↓ HbA1c ↓ total cholesterol, ↓ total protein	[198]
Resveratrol	Clinical trial: 192 diabetic patients (20–65 years old)	40 and 500 mg/day for 6 months	↓ glucose ↓ HbA1c ↓ HDL cholesterol ↓ insulin resistance	[199]
Cajanonic acid A	In vivo: Sprague-Dawley rats (n = 6 in each group)	15, 30 and 60 mg/kg for 24 h	↓ PPAR-γ ↓ PTP1B	[213]
Hydrangeic acid	In vivo: KK-A^y^ mice (n = 6) and C57BL/6 mice (n = 7)	200 mg/kg for 2 weeks	↓ glucose ↓ triglycerides ↓ free fatty acids	[214]
Hydrangeic acid	In vitro: 3T3-L1 cells	3–100 μM	↑ adiponectin ↑ 2-deoxyglucose uptake ↑ GLUT4 translocation	[214]
Lonchocarpene	In vivo: Swiss mice	100 mg/kg (one dose)	No effect on hyperglycemia	[205]
DPS	In vivo: Swiss mice	100 mg/kg (one dose)	↓ hyperglycemia	[205]

Abbreviations: ↑—increased; ↓—decreased; AMPK—5′ adenosine monophosphate-activated protein kinase; DPS—3,5-dimethoxy-4′-O-prenyl-trans-stilbene; GLUT4—glucose transporter type 4; HbA1c—glycated hemoglobin; HDL—high-density lipoprotein cholesterol; PPAR-γ—peroxisome proliferator-activated receptor-gamma; PTP1B—protein tyrosine phosphatase 1B; SIRT1—sirtuin 1; T2DM—type 2 diabetes mellitus.

**Table 5:** List of tannins in connection with T2DM.

Tannins	Research Models	Applied Treatment	Significant Observations	References
Tannin fraction	In vivo: STZ-induced diabetic rats (n = 6 in each group)	Tannin fraction from *Ficus racemosa* 100 and 200 mg/kg for 30 days	↓ glucose ↓ total cholesterol, LDL cholesterol, triglycerides ↓ GPx ↑ SOD, CAT ↑ insulin, HDL cholesterol	[237]
Different tannins	In vitro: 3T3-L1 cells	Lagerstroemin (0.04 mg/mL) Flosin B (0.2 mg/mL) Stachyurin (0.2 mg/mL) Casuarinin (0.04 mg/mL) Casuariin (1.0 mg/mL) 2,3-(S)-hexahydroxydiphenoyl-α/β-D-glucose (0.5 mg/mL)	↑ insulin-like glucose uptake ↓ adipocyte differentiation	[238]
Lagerstroemin Casuarinin	In vitro: 3T3-L1 cells	Lagerstroemin (0.04 mg/mL) Casuarinin (0.04 mg/mL)	↓ adipocyte differentiation	[238]
Procyanidin	In vivo: STZ-induced diabetic rats (n = 6–7 in each group)	Grape seed procyanidin extract 250 mg/kg	↓ hyperglycemia	[239]
Procyanidin	In vitro: L6E9 myoblasts, 3T3-L1 cells	Grape seed procyanidin extract 100 mg/L	↑ glucose uptake	[239]
Procyanidin	In vitro: CHO-IR cells	Grape seed procyanidin extract 100 mg/L	↑ glucose uptake ↑ phosphorylation of p38 MAPK	[240]
Procyanidin B2	In vivo: Diabetic db/db mice (n = 8 in each group)	Grape seeds 30 mg/kg for 10 weeks	↓ hepatic lipid metabolism ↓ food intake ↓ body weights ↓ triglycerides, total cholesterol, free fatty acid	[243]
Procyanidin B2	In vitro: 3T3-L1 cells	Grape seeds 150 µg/mL for 48 h	↓ adipogenesis	[244]
Phlorotannins	Clinical trial: 23 participants	blend of brown seaweed (*Ascophyllum nodosum* and *Fucus vesiculosus*) InSea2^TM^ two 250 mg capsules	↓ insulin incremental area under the curve ↑ insulin sensitivity	[248]
Phlorotannins	In vivo: Male Wistar rats (n = 6–8 in each group)	blend of brown seaweed (*Ascophyllum nodosum* and *Fucus vesiculosus*) InSea2^TM^ 7.5 mg/kg	↓ postprandial blood glucose ↓ insulin secretion	[249]
Phlorotannins	In vivo: Male albino Wistar rats (n = 10 in each group)	*Cystoseira compressa* phlorotannin extract 60 mg/kg	↓ glucose ↓ total triglycerides ↓ total cholesterol ↑ antioxidant capacity ↑ insulin concentration	[250]
Phlorotannins	In vivo: Male Wistar strain rats (n = 10 in each group)	70% methanol extract of *Pelvetia babingtonii* de Toni 1000 mg/kg	↓ postprandial blood glucose	[251]

Abbreviations: ↑—increased; ↓—decreased; CAT—catalase; GPx—glutathione peroxidase; HDL—high-density lipoprotein; LDL—low-density lipoprotein; MAPK—mitogen-activated protein kinases; SOD—superoxide dismutase; STZ—streptozotocin.

**Table 6:** List of lignans related to T2DM.

Lignans	Research Models	Applied Treatment	Significant Observations	References
Lignan-rich diet	Clinical trial: 6547 diabetic patients (2882 men and 3665 women; 41.3 ± 14.6 and 39.0 ± 13.4 years, respectively)	from 1.6 to 9.1 mg/day	↓ risk of T2DM	[260]
Lignan-rich diet	Clinical trial: 201,111 non-diabetic participants	from 355.1 (330.2–396.9) to 459.9 (422.2–519.5) μg/day	↓ HbA1c levels ↓ C-reactive protein levels	[261]
Lignan-rich diet	Clinical trial: 3430 non-diabetic participants (1314 men and 2116 women)	from 0.42 (0.35–0.47) to 0.78 (0.73–0.88) mg/day	Non-significant effect on T2DM	[74]
Syringaresinol	In vitro: BV2 microglia cells	25, 50, 100 and 200 μM for 1 h	↓ TNF-α, IL-1β ↓ COX-2, iNOS	[263]
Sesamin	In vivo: Sprague-Dawley rats (carrageenan-induced model of inflammation), (n = 4 in each group)	50 and 100 mg/kg for 2 days	↓ TNF-α, IL-1β, IL-8	[264]
Secoisolariciresinol diglucoside	In vivo: C57BL/6 mice (n = 6 in each group)	200 mg/kg for 1 week	↓ IL-1β, IL-18, TNF-α ↓ NLRP1	[265]

Abbreviations: ↑—increased; ↓—decreased; COX-2—cyclooxygenase-2; HbA1c—glycated hemoglobin; IL-1β—interleukin-1 beta; IL-8—interleukin-8; IL-18—interleukin-18; iNOS—inducible nitric oxide synthase; NLRP1—NLR family pyrin domain containing 1; T2DM—type 2 diabetes mellitus; TNF-α—tumor necrosis factor alpha.

**Table 7:** The interactions of antidiabetic drugs with various plants and polyphenols obtained from them.

Drug or Drug Groups	Research Models	Applied Treatment	Significant Observations	References
metformin	In vitro: 3T3-L1 adipocytes	20 μM metformin (MET) + 25 μM hydroxycinnamic derivatives (HD, ferulic acid, p-coumaric acid, eugenol, chlorogenic acid, caffeic acid) for 4 h	↑ 2-DG in MET + HD vs. MET → synergistic effect	[276]
metformin	In vivo: STZ-induced diabetic rats (n = 5 in each group)	50 mg/kg MET + 40 mg/kg ferulic acid (FA) for 3 weeks	↓ glucose ↓ triglycerides ↓ total cholesterol in MET + FA vs. MET → synergistic effect	[168]
metformin	In vivo: STZ-induced diabetic rats (n = 6 in each group)	200 mg/kg MET + 25 and 50 mg/kg gallic acid (GA) for 2 weeks	↓ glucose ↓ α-amylase ↓ α-glucosidase ↑ CAT in MET + GA vs. MET → synergistic effect	[275]
metformin	In vivo: HFD and STZ non-alcoholic fatty liver disease rats (n = 10 in each group)	100 mg/kg MET + 100 mg/kg malvidin (MAL) for 12 weeks	↓ glucose ↓ insulin resistance ↓ LDL cholesterol ↓ total cholesterol ↓ IL-8, IL-6 in MET + MAL vs. MET → synergistic effect	[274]
metformin	In vivo: HFD/STZ-induced diabetic mouse (n = 8 in each group) In vitro: HepG2 cells	50 and 100 mg/kg MET + 50 and 100 mg/kg cyanidin-3-arabinoside (C3A) for 6 weeks 20 µmol/L MET + 20 µmol/L anthocyanins	↓ glucose ↓ insulin resistance in MET + C3A vs. MET → synergistic effect ↑ glucose consumption in MET + anthocyanins vs. MET → synergistic effect	[118]
metformin	In vivo: HFD/STZ-induced diabetic rats (n = 6 in each group)	200 mg/kg MET + 100 mg/kg epigallocatechin-3-gallate (EGCG) for 28 days	↓ glucose ↓ cortisol → synergistic effect	[280]
metformin	In vitro: diabetic human skeletal muscle myoblasts	50 µg/mL MET + 150 µg/mL *Kalanchoe pinnata* extracts (quercetin, kaempferol, apigenin, epigallocatechin gallate, avicularin) for 72 h	↑ GPx ↑ CAT ↑ SOD in MET + *K. pinnata* vs. MET → synergistic effect	[273]
metformin	In vitro: human embryonic kidney (HEK) cells and Caco-2 cells	10 μM MET + 10 μM epigallocatechin gallate (EGCG) for 4 h	↓ MET uptake in MET + EGCG vs. MET → antagonistic effect	[277]
metformin	In vivo: glucose-induced hyperglycemic mice (n = 5 in each group)	150 mg/kg MET + 15 mg/kg acarbose (ACR) + *Abelmoschus esculentus* (L.) extract (AEE), 30, 60, 90, 150 min	↑ glucose in MET + ACR + AEE vs. MET + ACR → antagonistic effect	[278]
thiazolidinediones	In vitro: 3T3-L1 adipocytes	20 μM thiazolidinediones (TZD) + 25 μM hydroxycinnamic derivatives (HD) for 4 h	↑ 2-DG in TZD + HD vs. TZD → synergistic effect	[276]
thiazolidinediones	In vivo: STZ-induced diabetic rats (n = 5 in each group)	10 mg/kg thiazolidinediones (TZD) + 40 mg/kg ferulic acid (FA) for 3 weeks	↓ glucose ↓ triglycerides ↓ total cholesterol ↓ SGPT, SGOT in TZD + FA vs. TZD → synergistic effect	[168]
thiazolidinediones	In vivo: STZ-induced diabetic rats (n = 9 in each group)	0.65 mg/kg pioglitazone (PIO) + 20 mg/kg resveratrol (RE) for 8 weeks	↓ glucose ↓ TNF-α, IL-6 in PIO + RE vs. PIO → synergistic effect	[281]
thiazolidinediones	In vivo: Tsumura Suzuki Obese Diabetes (n = 6 in each group)	10 mg/kg/day pioglitazone (PIO) + 100 mg/kg/day naringenin (NAR) for 4 weeks	↑ glucose in PIO + NAR vs. PIO → potential antagonistic effect	[282]
sulfonylureas	In vivo: STZ-induced diabetic rats (n = 5 in each group)	5 mg/kg glibenclamide (GLIB) or glimepiride 2 mg/kg (GLIM) + 500 mg/kg leaf extract of *Azadirachta indica* (AI) for 10 days	↑ glucose in GLIB + AI or GLIM + AI vs either GLIB or GLIM → antagonistic effect	[284]
sulfonylureas	In vivo: nicotinamide- and STZ-induced diabetic mice (n = 6 in each group)	5 mg/kg glibenclamide (GLIB) + 300 mg/kg ethanolic extract from *Annona cherimola* (EEA) for 8 weeks	↑ glucose in EEA + GLIB vs. GLIB → antagonistic effect	[285]
α-glucosidase inhibitors	In vivo: STZ-induced diabetic rats (n = 6 in each group)	25 mg/kg acarbose (ACR) + 25 and 50 mg/kg gallic acid (GA) for 2 weeks	↓ α-glucosidase ↑ CAT in ACR + GA vs. ACR → synergistic effect	[275]
α-glucosidase inhibitors	In vivo: nicotinamide- and STZ-induced diabetic mice (n = 6 in each group)	50 mg/kg acarbose (ACR) + 50 mg/kg rutin (RU) for 8 weeks	↓ glucose in ACR + RU vs. ACR → synergistic effect	[285]
α-glucosidase inhibitors	In vivo: Kunming mice	0.3, 1, 80 mg/kg acarbose (ACR) + 40, 80, 240 mg/kg baicalein (BAE), quercetin (QUE, concentration not specified), luteolin (LUT, concentration not specified) and (+)-catechin (CATE, concentration not specified)	↓ α-glucosidase ↓ postprandial blood glucose in ACR + BAE, ACR + QUE, ACR + LUT vs. ACR → synergistic effect ↑ α-glucosidase ACR + CATE vs. ACR → antagonistic effect	[286]
dipeptidyl peptidase-4 inhibitors	In vitro: human and rat liver microsomes	2.5 mg/kg alogliptin (ALO), 1 mg/kg saxagliptin (SAX), and 10 mg/kg sitagliptin (SIT) + 100 mg/kg resveratrol (RES) for 7 days	↑ ROS stability of ALO, SAX, RES → synergistic effect	[287]
dipeptidyl peptidase-4 inhibitors	In vivo: STZ-induced diabetic rats (n = 6 in each group)	10 mg/kg sitagliptin (SIT) *+* 400 mg/kg *Eugenia jambolana* (EJ) extract for 28 days	↓ glucose ↓ triglycerides ↓ total cholesterol in SIT + EJ vs. SIT → additive effect	[288]
SGLT2 inhibitors	In vivo: STZ-induced diabetic mice (n = 6 in each group)	50 mg/kg canagliflozin (CANA) + 300 mg/kg extract from *Annona cherimola* (EEA) and 50 mg/kg rutin (RU) for 8 weeks	↓ glucose in CANA + EEA and CANA + RU vs. CANA → synergistic effect	[285]

Abbreviations: ↑—increased; ↓—decreased; 2-DG—2-Deoxy-D-glucose; CAT—catalase; GPx—glutathione peroxidase; HFD—high-fat diet; IL-6—interleukin 6; IL-8—interleukin 8; LDL—low-density lipoprotein; ROS—reactive oxygen species; SOD—superoxide dismutase; SGOT—serum glutamate oxaloacetate transaminase; SGPT—serum glutamate pyruvate transaminase; STZ—streptozotocin; TNF-α—tumor necrosis factor alpha.
